# Level up the brain! Novel PCA method reveals key neuroplastic refinements in action video gamers

**DOI:** 10.1162/IMAG.a.1090

**Published:** 2026-01-16

**Authors:** Kyle Cahill, Mukesh Dhamala

**Affiliations:** Department of Physics and Astronomy, Georgia State University, Atlanta, GA, United States; Neuroscience Institute, Georgia State University, Atlanta, GA, United States; Tri-Institutional Center for Translational Research in Neuroimaging and Data Science (TReNDS), Georgia State University, Georgia Institute of Technology, and Emory University, Atlanta, GA, United States; Center for Behavioral Neuroscience, Center for Diagnostics and Therapeutics, Georgia State University, Atlanta, GA, United States

**Keywords:** action video games, neuroplasticity, visuomotor decision making, multimodal connectivity, principal component analysis, cognitive resource reallocation

## Abstract

Action video games (AVGs) provide an ecologically rich context for examining how sustained cognitive demands relate to behaviorally induced neuroplasticity. In this cross-sectional study, we show that the connectivity differences observed in long-term AVG players, referred to here as gamers, reflect neuroplastic refinements consistent with more efficient neural mechanisms for reducing visuomotor information surprise during decision making. To explain how such adaptations could unfold over time, we utilized the Cognitive Resource Reallocation (CRR) framework, defined as the dynamic redistribution of metabolic and functional resources to support behaviorally relevant neuroplastic adaptation under repeated, demanding task conditions. Using a novel region-cumulative principal component analysis (rcPCA) approach applied to previously published data, we identified the subset of brain regions that best explain inter-subject variability, thereby improving statistical power and reducing the burden of multiple comparisons. Our results suggest that long-term engagement with AVGs may promote more efficient visuomotor decision-making strategies through both top–down cognitive clarity, enabling unobstructed transformation of learned value into goal-directed action, and bottom–up motor readiness, enhancing rapid and skillful action selection. These converging adaptations reduce internal conflict, mitigate uncertainty, and enable more effective translation of sensory input into coherent motor output, supporting the broader view that repeated cognitive challenge is associated with perturbations in neurodynamic equilibria that coincide with functional reorganization and enhanced cognitive ability.

## 1 Introduction

Video games provide a unique window into how complex, interactive environments shape the human brain. As one of the most widely consumed and cognitively engaging forms of entertainment globally (Media & Entertainment: Video Games Sector, 2024), video games have become a natural platform for studying experience-dependent neuroplasticity (Brilliant et al., 2019; Palaus et al., 2017). Action video games (AVGs) stand out for their ability to consistently elicit high cognitive load (Bediou et al., 2023). They place players in fast-paced, sensory-rich environments that demand sustained attention, rapid visuomotor integration, and split-second decision making.

Long-term exposure to AVGs has been associated with improved response times (RTs) (Jordan & Dhamala, 2022b), enhanced attentional control (Bavelier & Green, 2019; Green & Bavelier, 2012; Mancarella et al., 2022), more efficient sensorimotor learning and transformation (Gozli et al., 2014; Granek et al., 2010), and cognitive flexibility (Glass et al., 2013). These behavioral enhancements have also been shown to transfer to real-world domains such as surgery (Lynch et al., 2010), driving (Howard et al., 2023), improved reading ability (Bertoni et al., 2024; Tulloch & Pammer, 2019), enhanced visual function in adults with amblyopia (Vedamurthy et al., 2015), and military training (Orvis et al., 2010).

Existing AVG neuroimaging studies have reported changes in cortical thickness, gray matter volume (Kühn & Gallinat, 2014; Kühn, Gleich, et al., 2014; Kühn, Lorenz, et al., 2014), and white matter integrity in AVG players (hereafter referred to as “gamers”) (Cahill et al., 2024; Lewandowska et al., 2022), as well as altered activation in frontoparietal (Bertoni et al., 2024; Focker et al., 2018; Huang & Cheng, 2022), visual-attention (Bavelier et al., 2012; Cahill et al., 2024; Jordan & Dhamala, 2022a; Tulloch & Pammer, 2019), and multisensory circuits modulated by the insula (Bertoni et al., 2024). Notably, even a single session of action-like gameplay has been shown to causally engage salience processing (Franceschini et al., 2025).

However, with respect to AVGs, few studies have integrated functional, structural, and directed connectivity and have tested whether these neural differences align with measurable behavioral outcomes. Furthermore, a parsimonious explanation at the level of large-scale brain connectivity for how AVGs could plausibly drive these improvements remains elusive (Bediou et al., 2023; Brilliant et al., 2019; Huang & Cheng, 2022; Kühn et al., 2019; Palaus et al., 2017).

Neuroplasticity refers to the brain’s capacity to reorganize its structure and function in response to sustained cognitive or environmental demands. Although its molecular and cellular mechanisms such as Hebbian learning, long-term potentiation, synaptic pruning, and homeostatic regulation are well characterized at the mesoscale (Puderbaugh & Emmady, 2024), the field still lacks a unifying account of how these mechanisms cooperate to produce coordinated, large-scale network adaptations.

Cognitive Resource Reallocation (CRR) is proposed as a plausible systems-level framework linking these mesoscale processes to macroscale brain organization (Cahill & Dhamala, 2025). CRR builds upon prior work suggesting that the brain optimizes cognitive performance by reallocating functional and metabolic resources toward anatomically plausible, behaviorally relevant circuits. Resource reallocation has been thought to enable a neural system to more effectively meet strenuous or salient task demands (Barbot et al., 2021; Buhusi & Meck, 2009; Li et al., 2025) and respond to pathologies, such as in Alzheimer’s disease (Becker et al., 1996) that would allow the system to reduce prediction error under the Free Energy Principle (Friston, 2010; Friston & Kiebel, 2009) by enhancing behaviorally relevant neural processing while maintaining stationary action and adherence to thermodynamic constraints.

In the context of CRR with respect to AVG play, the brain may reinforce a baseline level of visuomotor efficiency, essential for maintaining moderate gameplay success. Rather, it minimizes response time in order to reduce its own action reinforcing task-relevant circuits necessary for AVG play while down regulating degenerate or inefficient ones. Action video games impose rapid, high-stakes decisions in sensory-rich, time-pressured environments where success depends on precise perception–action coupling. With repeated exposure, the strenuous demands of AVGs are expected to align neural activity along AVG task-relevant circuits. This study does not aim to prove CRR, nor does it attempt to quantify visuomotor surprise but rather examines whether the observed changes in AVG players may reflect task-relevant connectivity patterns that align with CRR as a plausible explanation.

Our hypothesis in this study is that long-term action video game play reflects brain connectivity differences between gamers and non-gamers that align with greater feedforward processing, motor readiness, improved anticipation, integration, and transformation of high-value visual cues into goal-directed action in gamers which are cognitive functions that directly support visuomotor decision making and are essential for playing AVGs. While direct causal claims cannot be made from this study’s cross-sectional design, this configuration would indicate a more efficient, goal-directed neural organization when making visuomotor decisions. In contrast, non-gamers may rely more on compensatory uncertainty-reducing feedback loops, early visual processing for object discrimination (e.g., of dot trajectories), and patterns consistent with less efficient visuomotor transformations.

In tasks like our moving-dots paradigm (50/50 outcomes) (Jordan & Dhamala, 2022b), we suspect gamers anticipate both possibilities more readily, consistent with greater cognitive flexibility and attentional control (Bavelier et al., 2018; Glass et al., 2013). These adaptations would be reflected in connections that promote feedforward, goal-directed action selection and the integration of scene-specific visual contextual cues (Green & Bavelier, 2006, 2007; Wu & Spence, 2013; Zinchenko et al., 2022), thereby resolving visuomotor uncertainty more efficiently. The absence or reversal of these predictions would falsify the hypothesis.

To test our hypothesis, we analyzed multimodal neuroimaging data from gamers and non-gamers who participated in a modified moving-dots task designed to probe visuomotor decision making (Jordan & Dhamala, 2022b). Our previously published behavioral results revealed that long-term action video gamers exhibited faster response times (~190 ms) with marginal gains in accuracy (~2–3%) compared with non-gamers. In decision-making literature, speed–accuracy trade-offs are typically accepted as the norm (Derosiere et al., 2021; Drugowitsch et al., 2015). Therefore, this effect requires a parsimonious explanation. We analyzed structural connectivity (SC) using 13 different SC measures provided by DSI Studio (Yeh, 2025), functional connectivity (FC), and directed time-domain Granger causality (dFC) in this study. Furthermore, we extracted submatrices of the dFC data into “sender” (outflow) and “receiver” (inflow) contributions, reflecting the directionality of influence between brain regions. The “total” dFC influence represents the combined interaction between these regions, incorporating both sender and receiver contributions. Finally, in addition to undirected and directed connectivity analyses, we examined combined structure–function measures including SFC undirected coupling and SdFC sender-mode coupling to explore alignment between a region’s structural substrate and its capacity for equivalent functional load, such as undirected synchrony (SFC) or outbound influence (SdFC sender).

Whole-brain connectivity matrices are high dimensional, comprising tens of thousands of pairwise connections, which posed significant challenges. Initial attempts to isolate meaningful whole-brain functional effects using conventional statistical thresholding yielded an overwhelming number of results across the AAL3 atlas’ 166 × 166 pairwise connections (Rolls et al., 2020), with no single principled method to distinguish signal from noise. Assessing all directed connections among 166 regions of the AAL3 atlas yields over 27,000 comparisons in anatomically unconstrained dFC data and over 13,000 comparisons in anatomically unconstrained symmetric FC data, despite a modest sample of 47 recruited participants, which severely diminished statistical power.

To address this challenge, a complementary data-driven PCA-based approach (Mwangi et al., 2014) was developed to sweep across the entire dataset and identify the strongest sources of inter-subject signal variance. Unlike traditional PCA approaches that reduce time series or select top ROIs from early components, the region-cumulative PCA (rcPCA) method utilizes cumulative variance-weighted contribution scores across all components, up to a defined cumulative explained variance threshold. This balances the goal of capturing meaningful inter-subject variation while excluding spurious components that reflect noise or isolated variance.

Using this novel region-cumulative principal component analysis (rcPCA) approach applied to previously published data (Jordan & Dhamala, 2022b), we identified key brain regions that explain inter-subject variability, improving statistical power by isolating the most informative regions and reducing the burden of multiple comparisons.

## 2 Materials and Methods

### 2.1 Participant data

A total of 47 right-handed participants were recruited for this study, including 28 action video game players (gamers; 4 female) and 19 non-gamers (12 female). The groups were age matched (gamers: 20.6 ± 2.4 years; non-gamers: 19.9 ± 2.6 years). Participants were classified as “gamers” if they reported playing at least 5 h per week of one or more action video game genres—including First-Person Shooter (FPS), Real-Time Strategy (RTS), Multiplayer Online Battle Arena (MOBA), or Battle Royale (BR)—consistently over the past 2 years. Non-gamers reported playing less than 30 min of video games per week over the same period. All participants passed the Ishihara color vision test to confirm normal color perception.

A modified left–right moving-dot (MD) task was used to probe visuomotor decision making and assess group differences in response time and accuracy (Jordan & Dhamala, 2022b). As reported in previously published behavioral data, gamers responded significantly faster and slightly more accurately than non-gamers. In the general condition, gamers responded significantly faster than non-gamers (930 ± 430 ms vs. 1120 ± 490 ms, *p* = 2.05 × 10^−70^), with an average response time advantage of 190 ms. They also exhibited significantly higher task accuracy (95.3% ± 3.9% vs. 93.0% ± 5.6%, *p* = 0.0008), reflecting a 2.2% improvement in overall performance accuracy (Jordan & Dhamala, 2022b).

After preprocessing and quality control (QC), the final sample size varied slightly by modality. Final Ns differed solely due to preprocessing QC (e.g., excessive head motion) and behavioral data completeness; analyses involving behavior required complete response time (RT) and accuracy data.

Functional and directed functional connectivity (FC and dFC) analyses included 43 participants (25 gamers and 18 non-gamers), while brain–behavior correlations using FC and dFC included 41 participants (24 gamers and 17 non-gamers). Structural connectivity (SC) analyses included 46 participants (27 gamers and 19 non-gamers), with 44 participants (26 gamers and 18 non-gamers) used in SC brain–behavior analyses. Finally, SC–FC and SC–dFC coupling analyses included 42 participants (24 gamers and 18 non-gamers), and the corresponding brain–behavior coupling analyses included 40 participants (23 gamers and 17 non-gamers). To confirm eligibility and assign participants to the appropriate group, a questionnaire was administered assessing video game genre and play frequency over the past 2 years. Sensitivity analysis indicated the study had 80% power to detect effects ≥ *d*≈0.84–0.90 across modalities. All participants passed the Ishihara Test for Color Deficiency and completed informed consent and health screening forms before data collection. The study was approved by the institutional review boards of Georgia State University and the Georgia Institute of Technology, both located in Atlanta, Georgia.

### 2.2 MRI data

#### 2.2.1 Data collection, scanning, and tractography protocols

Whole-brain structural and functional MR imaging was conducted on a 3T Siemens Magnetom Prisma MRI scanner (Siemens, Atlanta, GA, USA) at the joint Georgia State University and Georgia Institute of Technology Center for Advanced Brain Imaging, Atlanta, GA, USA. High-resolution anatomical images were acquired using a T1-MEMPRAGE scan sequence for voxel-based morphometry and anatomical reference. The acquisition parameters were as follows: TR = 2530 ms, TE1-4 = 1.69–7.27 ms, TI = 1260 ms, flip angle = 7°, and voxel size = 1 mm × 1 mm × 1 mm.

Diffusion-weighted imaging (DWI) data were collected using a multi-shell diffusion scheme with b-values of 300, 650, 1000, and 2000 s/mm², corresponding to 4, 17, 39, and 68 diffusion-encoding directions, respectively. One non-diffusion-weighted (b = 0) volume was also included. The acquisition was performed using a single-shot echo-planar imaging (EPI) sequence with anterior-to-posterior (AP) phase encoding. Each diffusion volume consisted of 60 axial slices acquired with a 2 mm isotropic resolution (slice thickness = 2 mm, in-plane resolution = 2 × 2 mm), and the field of view (ReadoutFOV) was 220 mm. The total scan duration was approximately 6.5 min. Acquisition parameters for the diffusion imaging included TR = 2750 ms and TE = 79 ms.

Following data acquisition, the diffusion data were reconstructed in the MNI space using q-space diffeomorphic reconstruction (QSDR) (Yeh & Tseng, 2011) to obtain the spin distribution function (SDF) (Yeh et al., 2010) in DSI Studio (Version Hou, 2024). A diffusion sampling length ratio of 1.25 was applied, with the output resolution in diffeomorphic reconstruction set to 2 mm isotropic. The tensor metrics were then calculated.

For fiber tractography, a deterministic fiber tracking algorithm (Yeh et al., 2013) was used, incorporating augmented tracking strategies (Yeh, 2020) to improve reproducibility. The quantitative anisotropy (QA) threshold was set to 0.12, and the angular threshold was set to 60 degrees. The step size was 1.00 mm, and tracks shorter than 10 mm or longer than 400 mm were discarded. A total of 5 million tracts were calculated for each subject. Shape analysis was conducted to derive shape metrics for the tractography (Yeh, 2020).

For full reproducibility, the parameter ID used in DSI Studio to configure these settings is 8FC2F53D9A99193Fba3Fb803Fcb2041bC843404B4Cca01cbaCDCC4C3Ec. This ID allows others to load the exact settings and parameters used in our analysis, ensuring that the tractography and other metrics can be reproduced using the same configurations.

The functional imaging was performed using a T2-weighted gradient echo-planar imaging (EPI)* sequence during the behavioral tasks. Four functional runs were acquired with the following parameters: TR = 535 ms, TE = 30 ms, flip angle = 46°, and voxel size = 3.8 mm × 3.8 mm × 4 mm. The field of view was 240 mm, and 32 slices were collected in an interleaved order with a slice thickness of 4 mm. A total of 3440 brain images were acquired during task performance.

#### 2.2.2 fMRI preprocessing pipeline

The preprocessing pipeline for functional MRI data combined tools from AFNI (Cox, 1996; Cox & Hyde, 1997), FSL (Jenkinson et al., 2012; Smith et al., 2004; Woolrich et al., 2009), and MRtrix3 (Veraart et al., 2016) to ensure high-quality data for subsequent analysis (Adhikari, Jahanshad, Shukla, Glahn, Blangero, Fox, et al., 2018; Adhikari, Jahanshad, Shukla, Glahn, Blangero, Reynolds, et al., 2018; Adhikari et al., 2019). The process began with denoising the fMRI data to reduce noise from physiological artifacts such as head motion and scanner drift, using MRtrix3’s dwidenoise command. This step resulted in denoised datasets for each run of the fMRI data. Following this, motion correction was performed using AFNI’s 3dvolreg, which registers each volume of the fMRI data to a reference volume within the session. After motion correction, the data were aligned to the MNI space using FSL’s FLIRT tool, ensuring that all data were in a common standard space for group-level comparisons.

To remove any motion-related artifacts, outlier detection was carried out using AFNI’s 3dToutcount, which computes the fraction of outlier voxels in each volume. A censoring procedure was then applied, excluding volumes where the fraction of outlier voxels exceeded a predefined threshold (0.1). Despiking was performed with AFNI’s 3dDespike, which removed brief, spurious signal fluctuations, or “spikes,” from the data. Following despiking, slice timing correction was applied using 3dTshift to ensure temporal alignment across slices in each volume.

Time series were then extracted from predefined brain regions based on the AAL3 parcellation using AFNI’s 3dROIstats, which computes the average signal within each region of interest (ROI). These time series were saved as text files for further analysis. Signal-to-noise ratio (SNR) for each run was computed using AFNI’s 3dTstat to calculate the mean signal and 3dTproject to compute the standard deviation of the noise. Additionally, global correlation averages (GCOR) were computed to assess overall data quality.

The degree of spatial blurring in the data was estimated using FSL’s 3dFWHMx, which calculates the full width at half maximum (FWHM) of the data’s spatial blurring. Following this, an extents mask was created using AFNI’s 3dmask_tool to identify valid brain regions with usable data across all volumes. The data were then registered to the MNI template (ENIGMA Template) using AFNI’s @auto_tlrc, which applies a transformation matrix to warp each subject’s data into standard space for group-level analysis.

To further improve data quality, principal component analysis (PCA) was applied using AFNI’s 3dpc to remove non-neuronal signals, such as global signal fluctuations and motion-related noise. PCA regressors were generated from ventricular and brain regions and were used in subsequent regression analysis to remove unwanted variance from the data. Finally, the processed datasets were reviewed for quality control, and any remaining temporary files were removed to prepare the data for further analysis.

After the AFNI and FSL preprocessing steps, additional processing was performed in MATLAB to further refine the data for task-based analysis. This included outlier correction, where extreme values in the time series that exceeded 5 standard deviations were identified and corrected by local linear interpolation. Detrending was applied to remove any linear trends from the data using MATLAB’s detrend function, ensuring that any slow drifts in the signal did not affect subsequent analyses. The time series data were then parsed by behavioral condition and time block, creating condition-specific time series data for each subject. This allowed for a more detailed analysis of brain activity that aligned with specific experimental conditions. The preprocessed time series data for each region of interest (ROI) in the AAL3 atlas were stored in structured files and saved for subsequent analysis.

### 2.3 Atlas selection and AAL3 parcellation

**Fig. 1.**
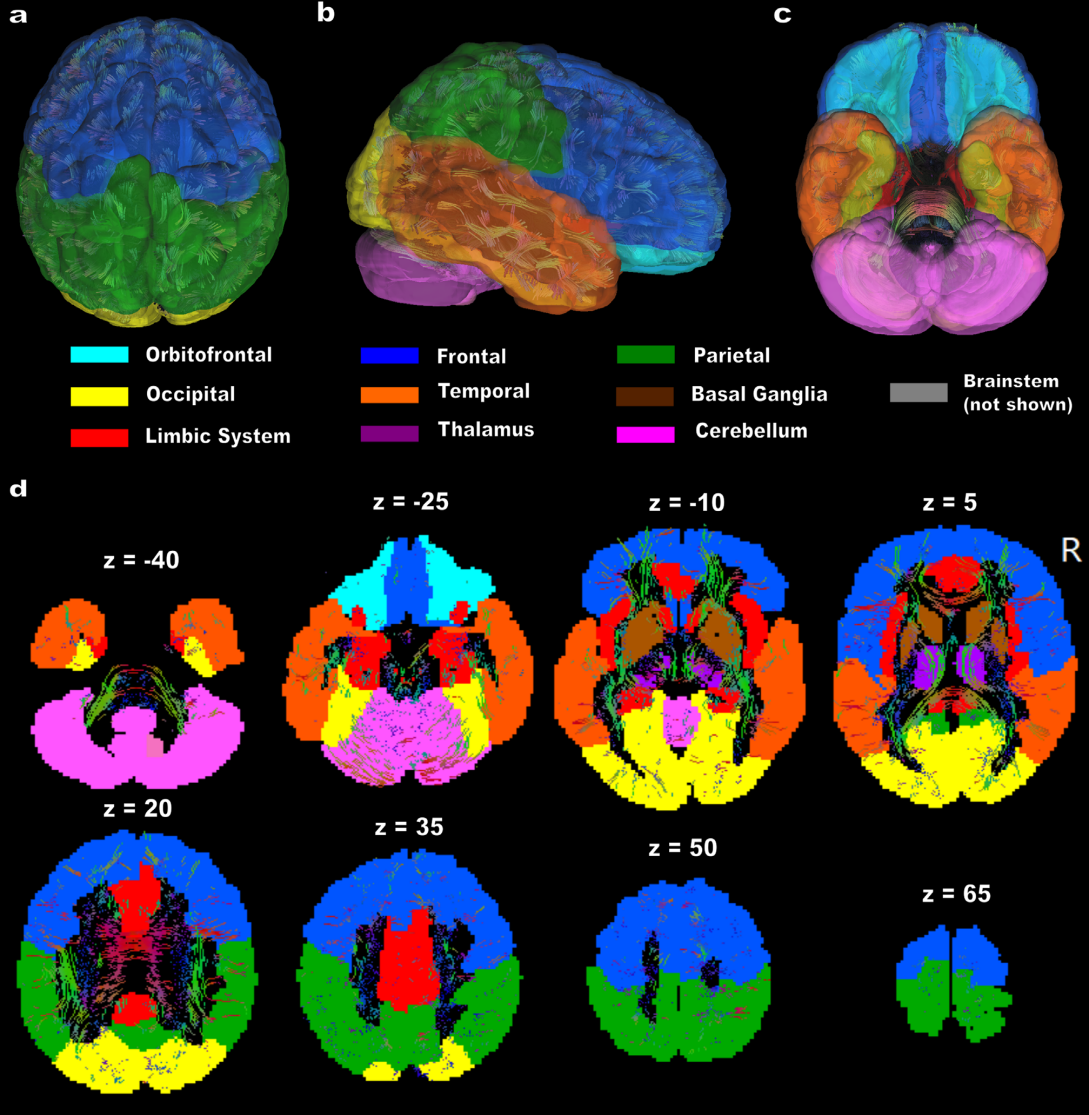
AAL3 Atlas Parcellation Categories for Connectivity Analysis. Visualization of the AAL3 atlas with anatomically grouped parcellations used in the connectivity analysis. (a) Superior view, (b) right lateral view, (c) inferior view. (d) Axial slices illustrate the parcellation structure along the z-axis. Colors correspond to distinct anatomical groups. The brainstem (gray) is not shown but is included in the analysis.

For the functional and structural connectivity analysis, the Automated Anatomical Labeling 3 (AAL3) atlas was selected due to its widespread use and strong effect sizes in capturing brain structure–function relationships, especially when compared with other well-known atlases (Revell et al., 2022). The AAL3 atlas includes 166 parcellations, with critical task-relevant regions such as the orbitofrontal cortex, cerebellum, and thalamic nuclei, which are particularly relevant for video game studies investigating neural processes underlying cognitive functions such as visuomotor decision making. This makes it highly suitable for whole-brain analysis in our study (Rolls et al., 2020). For better visual clarity and interpretability, we organized the regions of the AAL3 atlas into clear subdivisions Orbitofrontal, Occipital, Limbic System, Frontal, Temporal, Thalamus, Parietal, Basal Ganglia, Cerebellum, and Brain Stem (Fig. 1a) based on their known anatomical locations, while preserving individual regions in our analysis as displayed in Supplementary Figure S1. As the brain slices progress (Fig. 1b), the organization of these regions becomes more apparent, revealing how these anatomical structures are spatially arranged. This clear organizational structure aids in interpreting the results of our analysis.

### 2.4 rcPCA-based ROI selection for connectivity and structure–function coupling analysis

#### 2.4.1 Connectivity and coupling data

After preprocessing, functional connectivity (FC) was computed using pairwise Pearson correlations across the full set of parcellated time series (AAL3 regions). Directed functional connectivity (dFC) was estimated using pairwise time-domain Granger causality, following the procedure outlined in Dhamala et al. (2008). The evaluation of TGC was conducted in the frequency band in the range between $f_{1}$ = 0.05 Hz and $f_{2}$ = 0.9 Hz, with a sampling rate of 1.87 Hz ($TR^{-1}$ ).

The appropriate model order for the TGC analysis was determined by minimizing the spectral difference between the Granger-generated time series and the original signal, while maintaining sensitivity to trial-specific dynamics governed by the trial duration and the repetition time (TR). To preserve this sensitivity, the model order was constrained such that it did not exceed the number of time points within a trial. The maximum allowable model order, denoted ${\text{mo}}_{\text{max}},\;$ is given by

$${\text{mo}}_{\text{max}}=\frac{{\text{T}}_{\text{trial}}}{\text{TR}},{\text{ where T}}_{\text{trial}}\text{ is the inter}-\text{trial interval}.$$

The model order of 5 was selected as it best minimized the spectral difference in our dataset while preserving the sensitivity required to accommodate trial-specific GC influences. GC matrices were computed between the time series of the AAL3 brain regions, using this optimal model order, which effectively became our directed functional connectivity measure for this study.

For structural connectivity (SC) analysis, diffusion-weighted imaging data were processed to derive a structural connectivity matrix based on deterministic tractography using DSI-Studio. This process involved the use of q-space diffeomorphic reconstruction (QSDR) (Yeh, 2020; Yeh et al., 2010) and a deterministic fiber tracking algorithm (Yeh et al., 2013) to estimate the structural connectivity between brain regions. The resulting matrix quantified the number and strength of the structural connections between each pair of brain regions. To assess the degree of alignment between anatomical structure and functional signaling, structure–function coupling (SFC) metrics were computed by correlating structural connectivity (SC) matrices with both undirected functional connectivity (FC) and directed functional connectivity (dFC) (Fotiadis et al., 2024). All matrices were aligned using the AAL3 parcellation to ensure consistency across modalities.

The coupling between SC and FC (SFC) was computed by extracting, for each ROI pair, the corresponding values from the SC and FC matrices. Pearson correlation was then used to quantify the strength of association between anatomical connectivity and functional co-activation across subjects. The same approach was used to compute coupling between SC and directed functional connectivity (SdFC), where each subject’s SC matrix was compared against sender-mode Granger causality matrices. Although SC is symmetric and undirected, the correlation was performed by holding the row index constant and correlating each region’s structural projection pattern with its outgoing directed functional influences. This resulted in a region-wise measure of SdFC coupling, reflecting how well the structural architecture of a region supports its role as a functional sender.

Both intra-regional and pairwise coupling values were computed; however, to maintain a focused and interpretable analysis in the current study, we limited our exploration of structure–function coupling to intra-regional sender-mode values. This approach allowed us to establish a clean, region-specific profile of structure–function alignment. Future work may extend this framework to include full pairwise coupling analyses as a means of capturing distributed patterns of structural–functional integration.

#### 2.4.2 rcPCA-based ROI selection

To identify the most informative regions of interest (ROIs) across structural and functional connectivity domains, we developed a data-driven, region-cumulative principal component analysis (rcPCA) method for dimensionality reduction. The rcPCA approach decomposes subject-level connectivity matrices into orthogonal principal components and derives regional contribution scores by quantifying each ROI’s influence on the structured variance of the dataset. All analyses were implemented in MATLAB using custom scripts developed for this study. This method adapts standard PCA protocols and interpretation techniques commonly used in the neuroimaging community, focusing on high-loading features per component to enable principled ROI selection and structured dimensionality reduction without sacrificing interpretability. To prioritize meaningful contributors while minimizing noise, we capped contribution accumulation at the top 20 ROIs per component. This strategy was chosen to avoid rank dilution from low-weight contributors, improve interpretability, and emphasize ROIs that consistently explain variance across components. Including all ROIs per component would risk amplifying weak or spurious contributions, particularly in sparser or asymmetric modalities such as structural or directed connectivity. While this approach may sacrifice global monotonicity with raw variance rankings, it enhances the salience of high-impact regions, aligning with our goal of identifying interpretable, behaviorally relevant ROIs.

For undirected connectivity data such as functional connectivity (FC), we first removed diagonal elements from each subject’s 166 × 166 ROI-wise matrix and reshaped the resulting 3D array into a 2-dimensional matrix of size n × E, where n is the number of subjects and E is the number of unique off-diagonal connections between ROIs. This matrix reshaping was handled by the script undirected_pca_analysis.m. This script then called our custom function run_pca.m that uses MATLAB’s built-in *pca()* function, which performs singular value decomposition (SVD) (Wall et al., 2002) on a mean-centered data matrix of size n × E. This function returns the component loadings, subject scores, and variance explained by each principal component. ROI-level contributions were used to interpret latent components, while subject-level scores served as PCA-derived features for potential classifier enrichment.

Finally, we identified the ROI contributions for each component by summing the absolute values of the PCA loadings associated with each region. For connectivity-wise decompositions (e.g., FC), loadings were reshaped into full ROI × ROI matrices with NaNs along the diagonal to exclude self-connections. The sum of absolute values across each row yielded a scalar contribution score per region, reflecting how strongly a given ROI influenced the variance captured by that component. For ROI-wise decompositions (e.g., structure–function coupling), each coefficient directly corresponded to a region, and the absolute values were taken as contribution scores. ROIs were ranked in descending order for each component and saved for downstream analysis.

To compute each region’s cumulative contribution to the explained variance, we used the script *undirected_pca_roi_contributions.m*. This script sets the number of top pca rois, cumulative variance percentage, and calls the custom function *pca*_*roi_contributions.m* which calculates the cumulative contribution of each roi to the user’s desired cumulative explained variance threshold which we set to 80% using a weighted sum set by the weights of each PC. By capturing 80% of the total explained variance across components, we retain the dominant patterns in the data that are most likely to reflect structured signal rather than noise, while discarding components that account for only a small portion of the variance which may be less interpretable and more sensitive to measurement error. This threshold also stabilizes the ranking of contributing ROIs across modalities and ensures that our contribution scores are derived from a meaningful, high-variance subspace rather than dominated by noisy low-variance dimensions. This procedure was carried out not only for FC, but also for all 13 categorically distinct structural connectivity (SC) metrics—(axial diffusivity (AD); streamline count; fractional anisotropy (FA); isotropy (ISO); mean diffusivity (MD); mean streamline length; Ncount; Ncount2; normalized quantitative anisotropy (NQA); non-restricted diffusivity imaging (NRDI); radial diffusivity (RD); restricted diffusivity imaging (RDI))—as well as for all 13 SFC and all 13 SdFC coupling matrices, using the same run_pca.m and ROI contribution framework. For definitions of each metric, see Table 1.

**Table 1.** Structural connectivity (SC) measures and definitions reflecting standard interpretations of diffusion and tractography measures from DSI Studio.

SC measure	Description
Axial diffusivity (AD)	Diffusion along the primary fiber axis; reflects axonal integrity.
Count	Raw number of tractography streamlines between regions.
Fractional anisotropy (FA)	Degree of directional water diffusion; higher values indicate organized fiber structure, broadly linked to myelination.
Isotropy (ISO)	Uniformity of diffusion in all directions
Mean diffusivity (MD)	Average diffusion in all directions; reflects overall water mobility.
Mean length	Average length of streamlines between ROIs
Non-restricted diffusion imaging (NRDI)	Measures extracellular diffusion; sensitive to edema or extracellular space.
Normalized count (Ncount)	Streamline count adjusted for ROI size and distance.
Normalized count (inverse-length weighted) (Ncount2)	Streamline count weighted by inverse length; emphasizes shorter, potentially more reliable tracts.
Normalized quantitative anisotropy (NQA)	Tracks the anisotropy of the principal fiber direction, normalized to background noise.
Quantitative anisotropy) QA	Signal intensity along the principal fiber orientation; related to tract integrity.
Radial diffusivity (RD)	Diffusion perpendicular to the main axis; associated with myelin integrity.
Restricted diffusion imaging (RDI)	Estimates intracellular diffusion; linked to axonal density

An ICA-based method for dimensionality reduction was initially considered, but rcPCA was ultimately developed as a more suitable alternative for this study’s goal of investigating how AVG experience shapes SC, FC, dFC, SFC, and SdFC (sender) profiles within a predefined anatomical atlas such as AAL3. Independent component analysis (ICA) is a powerful tool for uncovering distributed functional networks via blind source separation, using higher-order statistics such as kurtosis or negentropy, and has yielded valuable insights into large-scale brain organization (Calhoun et al., 2001, 2009). While ICA excels at detecting latent sources and identifying network-level structure, it does not natively support anatomically localized, region-level ranking. Techniques such as spatial template matching, dual regression, ICN labeling, and joint ICA each extend ICA’s utility, with template matching aiding anatomical alignment, dual regression estimating subject-level component expression, ICN labeling providing network-level categorization, and joint ICA enabling multimodal data fusion. However, none of these approaches cleanly resolve the core challenge of identifying which anatomical regions within a predefined structural atlas such as AAL3 contribute most meaningfully to inter-subject variability across connectivity modalities.

Each analysis followed the same pipeline: data reshaping, PCA decomposition, ROI-wise contribution scoring, and cumulative variance weighting, providing a unified approach to data-driven region selection across modalities.

For directed connectivity data, such as Granger-causal (dFC) matrices, we developed a parallel pipeline using the scripts *directed_pca_analysis.m* and *run_dpca.m*. Since directed matrices represent asymmetric interactions between regions (i.e., sender to receiver), we performed rcPCA separately for sender, receiver, and total connectivity modes. Directed connectivity matrices are square, where each element represents the strength of influence from one region (sender) to another (receiver). A companion script to *pca_roi_contributions.m*, titled *directed_roi_contributions_dpca.m*, was created to compute cumulative contributions in sender, receiver, and total modes. For each region, cumulative sender influence was calculated by holding the row index constant and summing across columns, whereas cumulative receiver influence was computed by holding the column index constant and summing across rows. Total contributions were obtained by summing both sender and receiver values for each region. These direction-specific contributions were then used to rank ROIs according to their influence in the structured variance of the directed connectivity data.

All outputs—including PCA loadings, explained variance, region rankings, and component-wise visualizations—were saved for each subject group and connectivity modality. In addition, the top ROIs, their corresponding AAL3 region labels, and all cumulative ROI contribution scores were saved to support downstream interpretation and reproducibility. After confirming the internal validity of our approach by comparing PCA-derived region rankings with raw variance rankings within each connectivity metric, these results were used in downstream analyses to identify high-variance ROIs for group comparisons and behavioral correlation testing.

Overall, this rcPCA framework offers a principled and scalable strategy for prioritizing informative brain regions in high-dimensional neuroimaging data. By reducing noise and redundancy while preserving structured variability, the method improves sensitivity to behaviorally relevant effects and significantly lowers the burden of multiple comparisons.

#### 2.4.3 Validation of rcPCA-derived region rankings

To evaluate the internal consistency and robustness of rcPCA, a multi-pronged validation was developed to test the derived regional rankings compared with the raw variance using a combination of rank correlation, permutation testing, and hypergeometric overlap statistics using a significance threshold of *p* < 0.001. This procedure was implemented in the script *validate_pca_variance.m* and carried out separately for each modality, including FC, dFC, SC, SFC, and SdFC (sender).

First, the regions were ranked according to their raw pre-PCA variance. This ranking was then compared with PCA-derived rankings using Spearman’s rank correlation across a range of top-k values, from 2 up to 166, reflecting the full resolution of the AAL3 parcellation. These tests provided converging evidence that the region-based cumulative PCA (rcPCA) method reliably identifies regions based on structured variance patterns, supporting the method’s selectivity and stability across modalities. The choice of k = 20 offered a practical compromise, large enough to capture meaningful connectivity patterns while remaining selective enough to highlight informative ROIs.

In the Spearman analysis, we calculated rank correlation coefficients between the top-k PCA-derived ROIs and the top-k raw-variance ROIs at each k. This approach was chosen for its robustness to non-normal distributions and its sensitivity to monotonic relationships. The correlation coefficient was calculated as

$$\rho =\frac{cov\left({\text{ranks }}_{raw},{\text{ ranks}}_{PCA}\right)}{\sigma_{\text{ranks,raw }}\sigma_{\text{ranks,PCA }}},$$

where ${\text{ranks }}_{\text{raw }}$ are the ranks of the raw data, $r{\text{anks }}_{\text{PCA }}$ are the ranks of regions based on the cumulative PCA-derived variance contributions, cov is the covariance between two ranked vectors, and σ is the standard deviation of ranks. The correlation was computed in MATLAB using the *corr()* function with the “Type”, “Spearman” option, which is given by

$$[\rho_{\text{k},},p_{k}]=\text{corr}\left(rank_{raw}\_k,\;ran{\text{k}}_{\text{PCA}}\_\text{k},\;‘Type’,\;‘Spearman’\right).$$

To assess whether the number of overlapping ROIs between the top-k PCA-selected and raw variance-selected regions could be attributed to chance, we used the cumulative distribution function of the hypergeometric distribution. This distribution models the probability of x or more successes (i.e., overlapping ROIs) when drawing two sets of size k from a population of N = 166 ROIs without replacement. The probability of observing at least x overlaps under the null hypothesis of random selection is given by the standard expression for hypergeometric *p*-value

$$p_{\text{hyper}}\left(x\right)=1-\sum_{i=0}^{x-1}\frac{\left(\begin{array}{c} k\\ i \end{array}\right)\left(\begin{array}{c} N-k\\ k-i \end{array}\right)}{\left(\begin{array}{c} N\\ k \end{array}\right)}$$,

where N is the total number of ROIs (166), k is the number of top-ranked ROIs selected in each set, and x is the observed number of overlapping ROIs between the PCA-based and raw variance-based rankings. This computation was implemented in MATLAB using the built-in hygecdf function and is given by (p_hyper = 1 - hygecdf(x - 1, N, k, k)).

Finally, a null distribution of random overlap values was generated by permuting the raw variance ROI rankings 10,000 times. For each value of k, the top-k ROIs were selected from each permutation, and their overlap with the PCA-selected top-k ROIs, denoted as x, was recorded. The empirical *p*-value was then computed as the proportion of permutations in which the number of overlapping ROIs was greater than or equal to x, relative to the total number of permutations

$$p_{\text{perm}}=\frac{\#\text{ of null overlaps }\geq x}{\text{num\_permutations}},$$

and the equivalent MATLAB statement used for this calculation is given by (perm_pvals(i) = mean(rand_overlaps >= overlap_counts(i))). This allowed us to assess whether the observed overlap between PCA-selected and raw variance-based regions exceeded what would be expected by chance.

All results, including overlap counts, *p*-values (Spearman, hypergeometric, and permutation), and rank correlations, were logged and plotted across k-values. Summary figures included correlation curves and significance levels as a function of k, bar plots comparing top-k PCA- and raw-variance ROI values, and permutation histograms and hypergeometric threshold overlays for observed overlap. Across all modalities, hypergeometric and permutation tests yielded exceptionally low *p*-values, often well below the *p* < 0.001 threshold. At higher values of k, both tests frequently reached the limits of machine precision. In such cases, MATLAB returned literal zero values due to numerical underflow, requiring the imposition of a floor at *p* = 1e-15. This indicated that the observed overlaps were so unlikely under the null distribution that their probabilities could not be accurately represented in double-precision floating-point arithmetic.

This provides compelling evidence that the overlap between rcPCA-derived and raw-variance ROI rankings was highly unlikely to occur by chance. They further validate the selectivity, robustness, and internal consistency of the proposed method. Notably, using cumulative contributions across all ROIs before selecting the top 20 restored the Spearman rank significance that was lost in the dFC receiver, SC, SFC, and SdFC modalities when only the top 20 ROIs per component were used during accumulation. These validation results confirmed that PCA-selected ROIs consistently overlapped with high-variance regions across modalities, supporting the interpretability, reproducibility, and robustness of the rcPCA selection method. For final visualizations, we used “n” in place of “k” to denote the number of top-ranked ROIs, as it provided a clear and intuitive shorthand in contexts where there was no conflicting N variable.

Furthermore, full convergence of rcPCA-derived ROI rankings with raw variance rankings, across all validation metrics, was observed within the range of k = 100–150. This included Spearman correlations approaching unity, permutation-based *p*-values below machine-level significance (*p* < 1e-15), and hypergeometric overlaps exceeding chance across all thresholds. Isotropy was the last measure to show full convergence, but did so by k = 90, making the k = 100–150 range a conservative benchmark for achieving maximal rank agreement. This defines a convergence ceiling for rcPCA-based variance decomposition in whole-brain neuroimaging data for the AAL3 atlas. The observed convergence window likely reflects the resolution of the AAL3 atlas’s 166 ROIs. While this range defines full convergence for this specific parcellation, the maximum k required for machine-level agreement is expected to scale proportionally with atlas dimensionality.

Another observation was that the permutation-based *p*-values exhibited a characteristic rebound at high k values (e.g., k ≥ 160), as the overlap between random samples and observed sets approached the full ROI space. This behavior reflects the design of permutation tests, which become less discriminative as sampling exhausts the comparison space. This plateau confirms that *p*-value inflation near the maximum ROI count is an expected property of null model behavior due to sampling saturation.

Together, these procedures confirm that the rcPCA-based ROI selection framework reliably identifies regions that meaningfully contribute to structured variance across modalities. The strong convergence across multiple statistical tests affirms that top-ranked ROIs are not artifacts of random variation, but rather reflect well-founded, mathematically principled selection criteria. In short, the method behaved exactly as intended, yielding high-variance-contributing, interpretable ROI candidates for downstream analysis.

#### 2.4.4 Group comparisons and brain–behavior relationships (rcPCA-derived ROIs)

To investigate group-level differences and behavioral relevance of connectivity across modalities, we used the top 20 rcPCA-derived ROIs from each modality-specific decomposition as filters. For each modality—functional connectivity (FC), directed connectivity (dFC; sender, receiver, and total), structural connectivity (SC), and coupling metrics (SFC and SdFC)—we extracted submatrices containing any connection involving a top-ranked ROI. These filtered matrices were used to run non-parametric group comparisons (Mann–Whitney U, rank sum (McKnight & Najab, 2010) tests) between gamers and non-gamers and Pearson brain–behavior correlations with response time (RT) as a behavioral measure.

False discovery rate (FDR) correction was applied independently within each connectivity modality to account for multiple comparisons during group comparisons. Two standard approaches were considered: the Storey–Tibshirani (ST) method (Storey & Tibshirani, 2003) (which estimates the proportion of true null hypotheses, π₀) and the Benjamini–Hochberg (BH) procedure (Benjamini & Hochberg, 1995) (which ranks *p*-values and applies a fixed step-up threshold). The choice between them was guided by both observed performance and the underlying assumptions of each method.

The ST method was deemed valid for functional connectivity (FC) data only. In all FC comparisons, the estimated *q*-values remained consistently less than or equal to the corresponding uncorrected *p*-values, indicating that the proportion of true nulls could be reliably estimated and that the method behaved as expected. These results suggest that Storey’s assumptions were met for FC—likely due to the dense, symmetric, and continuous nature of FC matrices, along with their relatively uniform distribution of connectivity values.

In contrast, Storey’s method was not considered reliable for the other modalities. In structural connectivity (SC), directed connectivity (dFC), and coupling metrics (SFC, SdFC), *q*-values were frequently observed to fall below their corresponding *p*-values despite *p*-values being well above the uncorrected significance threshold, a pattern inconsistent with valid FDR correction. This behavior indicated instability in π₀ estimation, likely due to skewed or zero-inflated distributions, sparse matrices, and directional asymmetries—conditions known to violate the assumptions behind Storey’s estimator.

To ensure robust and conservative control of false discoveries in these cases, the BH procedure was applied instead. BH does not rely on π₀ estimation and is more stable under non-ideal conditions. It was, therefore, used for all SC, SFC, SdFC, and dFC variants, where structural constraints or directional signal flow introduced potential sources of bias*.*

We computed Spearman rank correlations between response time (RT) and connectivity values for all connections involving the top 20 rcPCA-derived ROIs per modality. Tests used a two-tailed α = .05, and results were further filtered with an effect-size threshold of |r| ≥ 0.20 for interpretability. To avoid symbol overlap with ρ used in rcPCA validation, we denote the Spearman coefficient as r throughout the Results section and in Figure 7.

## 3 Results

The Region-Cumulative PCA (rcPCA) ROI selection strategy followed standard PCA-based practices (Jolliffe & Cadima, 2016; Lee et al., 2021) commonly used in neuroimaging studies (Lee et al., 2021; Mwangi et al., 2014), prioritizing high-loading features from each principal component (Kucukboyaci et al., 2014). Specifically, we selected the top 20 contributing ROIs per component to emphasize regions that most strongly explained structured variance while minimizing the inclusion of low-weight contributors. This approach aligns with established PCA interpretation techniques, which typically focus on dominant features from early components to maximize interpretability.

**Fig. 2.**
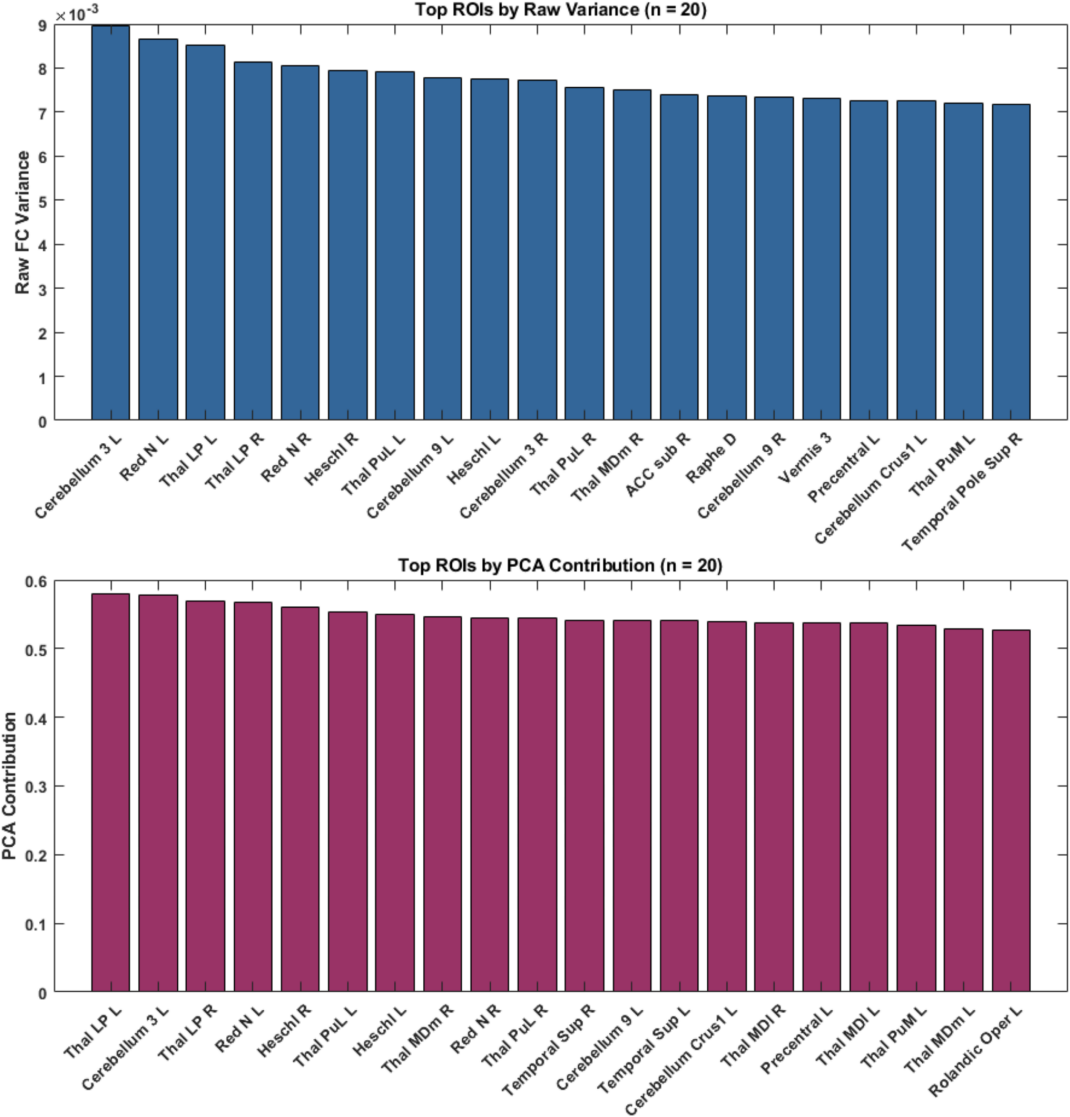
Top 20 ROIs by Across-Participant FC Variability (Raw Variance vs. Normalized rcPCA). Undirected functional connectivity (FC) matrices were compiled across participants. We applied region-cumulative PCA (rcPCA) and computed each ROI’s variance-weighted contribution using principal components up to 80% cumulative explained variance. The panels present the 20 top-ranked ROIs: top, raw FC variance (values scaled ×10−³) obtained directly from the data; bottom, rcPCA contribution (normalized) derived from covariance-weighted loadings integrated up to the 80% cumulative explained variance cutoff. The x-axis lists AAL3 ROI names, enabling direct comparison between rankings from raw-FC variance and those derived by rcPCA.

**Fig. 3.**
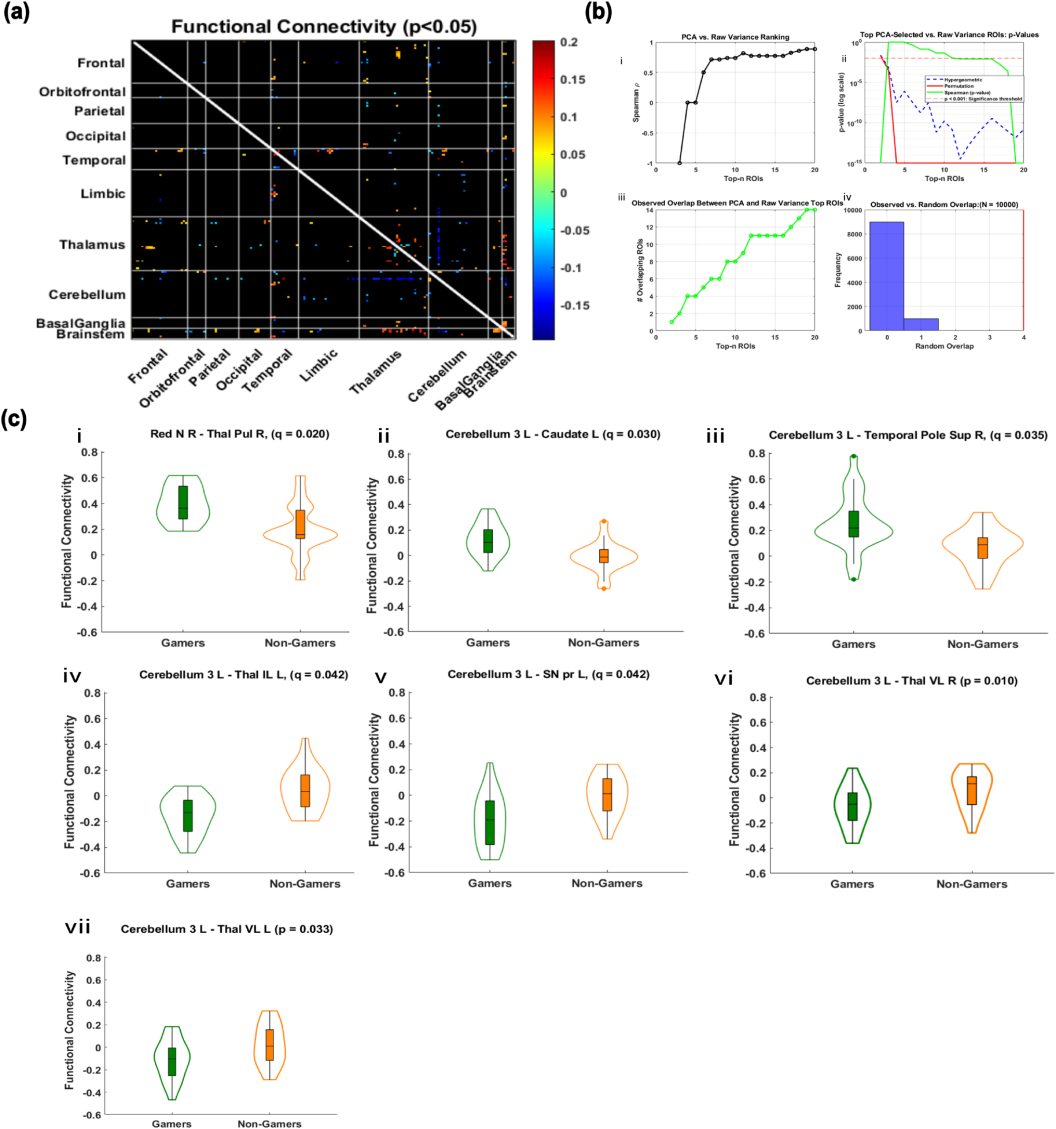
Functional Connectivity Differences Involving Top rcPCA ROIs. Multi-step analysis of functional connectivity (FC) group differences and PCA-based ROI selection. (a) Whole-brain FC matrix showing significant group differences (*p* < 0.05, uncorrected), organized by anatomical region. (b) Validation of the PCA-based method: (i) Spearman’s rank correlation (ρ) across top-n selections comparing PCA-weighted and raw variance-based rankings; (ii) comparison of statistical sensitivity using Spearman correlation, hypergeometric overlap *p*-values, and permutation-derived *p*-values between PCA- and raw-ranked ROIs; (iii) overlap between PCA- and raw-ranked ROIs increases systematically with top-n selections; (iv) PCA-selected ROI overlap exceeds chance across 10,000 permutations. (c) Violin plots illustrating significant FC differences (*q* < 0.05, FDR-corrected), involving top PCA-selected ROIs in cerebellar, subcortical, midbrain, thalamic, and temporal areas. Results (i-vii) are ordered from lowest to highest q-value; where q-values are not applicable, ordering is by lowest to highest p-value. Gamers are shown in green and non-gamers are shown in orange.

As shown in Figure 2, rcPCA-based ROI contributions exhibited strong correspondence with raw variance rankings across multiple brain connectivity metrics, including functional connectivity (FC). The Spearman correlation exceeded 0.85 across the top-ranked ROIs, with significance (*p* < 0.001) occurring rapidly as sample size (n) increased (see Fig. 3b). Validation of rcPCA-derived ROIs utilized a combination of rank correlation, permutation testing, and hypergeometric overlap statistics. Using a significance threshold of *p* < 0.001, this approach confirmed that the observed alignments between PCA-derived ROI rankings and raw variance were statistically robust and highly unlikely to occur by chance.

### 3.1 Group differences in functional and directed connectivity

#### 3.1.1 Group differences in functional connectivity

Whole-brain FC analysis revealed significant group differences (*p* < 0.05) between gamers and non-gamers, concentrated in thalamic, cerebellar, midbrain, and temporal regions (Fig. 3a). PCA-selected ROIs, validated in Figure 3b, showed robust effects following FDR correction. Gamers exhibited significantly stronger functional connectivity between the right red nucleus and the right pulvinar thalamic nucleus (*p* = 0.0061, *q* = 0.02, *d* = 0.85), and between left cerebellar lobule 3 and both the right superior temporal pole (*p* = 0.0014, *q* = 0.035, *d* = 1.12) and the left caudate nucleus (*p* = 0.00093, *q* = 0.03, *d* = 1.00). In contrast, non-gamers showed stronger connectivity between left cerebellar lobule 3 and the left intralaminar thalamic nuclei (*p* = 0.00085, *q* = 0.042, *d* = –1.26), the left substantia nigra pars reticulata (*p* = 0.002, *q* = 0.042, *d* = –1.10), and both the right and left ventrolateral thalamic nuclei (*p* = 0.01, *d* = –0.76; *p* = 0.03, *d* = –0.87, respectively). These effects reflect consistent, large-magnitude differences in functional connectivity across subcortical–cerebellar circuits. Absolute effect sizes were consistently large, ranging from *d* = 0.76 to 1.26 across significant connections.

Full statistical results, including all *p*-values, FDR-adjusted *q*-values, and Cohen’s *d* effect sizes for each comparison, are reported in Supplementary Figure S2.

#### 3.1.2 Group differences in directed functional connectivity

**Fig. 4.**
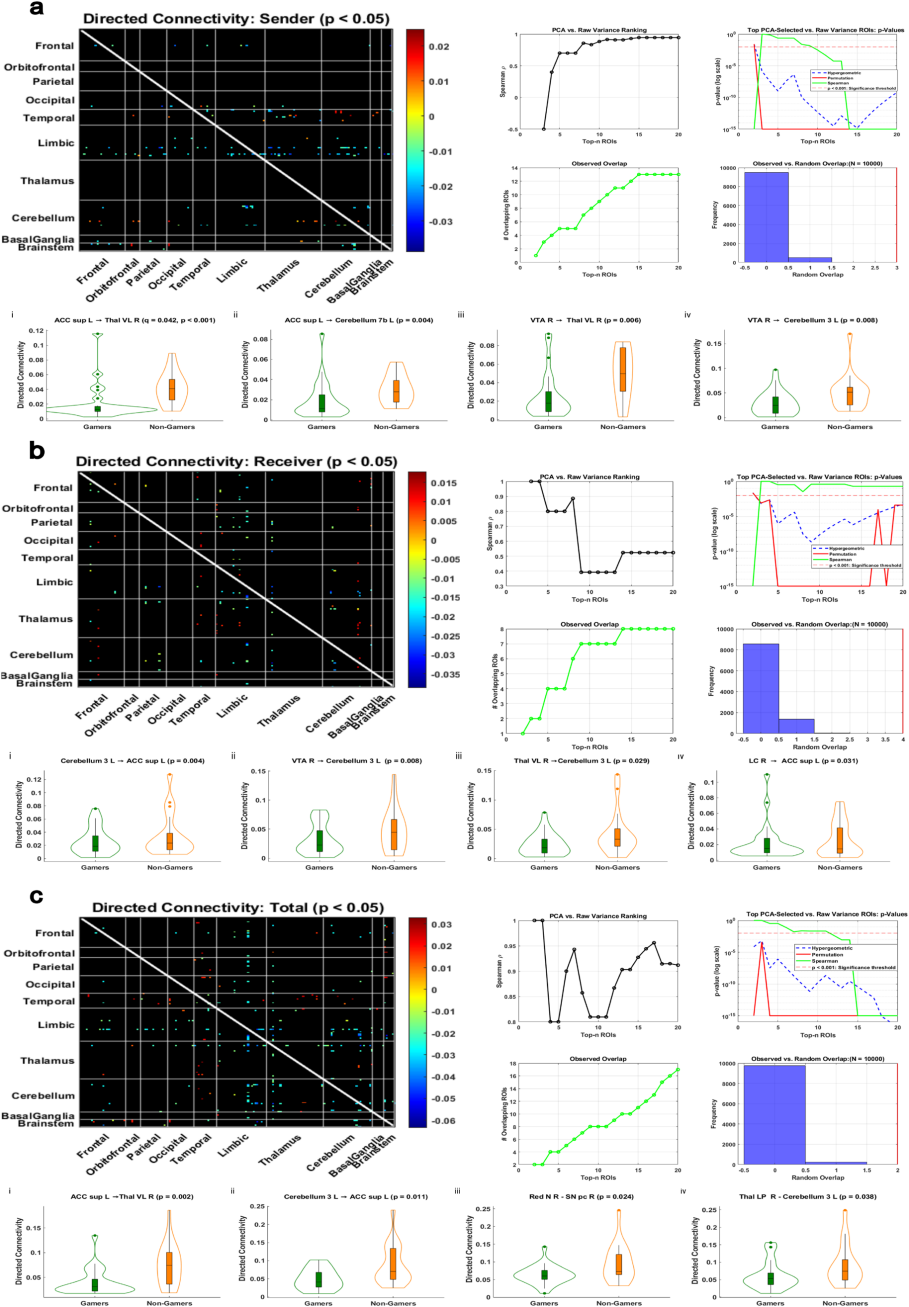
Directed connectivity differences involving top rcPCA ROIs. Group-level differences in directed functional connectivity (dFC) between gamers and non-gamers using PCA-based ROI selection. Each panel includes a full dFC matrix of significant group differences (p < 0.05), validation of PCA-based selection stability, and violin plots highlighting the strongest observed effects (p < 0.05). In the violin plots, gamers are shown in green and non-gamers in orange. Within each panel (a-c), results are ordered from lowest to highest p-value: (a) i-iv, (b) i-iv, and (c) i-iv. (a) dFC (sender): group differences in outgoing influence. A connection from the anterior cingulate cortex (ACC sup L) to the right thalamus (VL R) survived FDR correction (q < 0.05). Additional uncorrected effects were observed from VTA R to cerebellar and thalamic targets. (b) dFC (receiver): group differences in incoming influence. Effects were observed in Cerebellum 3 L receiving projections from VTA R and Thal VL R, and in ACC sup L receiving input from LC R. (c) dFC (total): group differences in direction-collapsed influence (sum of sender and receiver roles). Notable effects included connections involving ACC sup L, SN pc R, and Cerebellum 3 L.

Directed connectivity analyses using Granger causality revealed significant differences in directed functional connectivity (dFC) between gamers and non-gamers across sender, receiver, and total modes (*p* < 0.05, uncorrected). These effects were concentrated in midbrain, thalamic, cerebellar, and anterior cingulate cortex (ACC) regions (Fig. 4a–c). In sender mode, non-gamers exhibited stronger directed influence from the left supracallosal ACC to the right ventrolateral thalamus (*p* = 0.0003, *q* = 0.042, *d* = –0.91), the only connection that survived false discovery rate (FDR) correction. Additional uncorrected differences included stronger non-gamer outflow from the left ACC to the left cerebellar lobule 7b (*p* = 0.004, *d* = –0.65) and from the right ventral tegmental area (VTA) to both the right ventrolateral thalamus (*p* = 0.006, *d* = –0.96) and the left cerebellar lobule 3 (*p* = 0.012, *d* = –0.75).

In receiver mode, non-gamers showed greater inflow to the left supracallosal ACC from the left cerebellar lobule 3 (*p* = 0.004, *d* = –1.00) and the right locus coeruleus (*p* = 0.031, *d* = –0.83), while bilateral VTA and right thalamus also showed elevated input to the left cerebellum. Total mode effects reflected overlapping but distinct connectivity patterns, including stronger non-gamer influence from the left ACC to the right thalamus (*p* = 0.002, *d* = –0.98) and from the left cerebellar lobule 3 to the left ACC (*p* = 0.011, *d* = –0.87). Effect size magnitudes across modes ranged from *d* = 0.59 to 1.00, indicating robust directional asymmetries in dFC across groups. Full statistical results, including all *p*-values, FDR-adjusted *q*-values, and Cohen’s *d* effect sizes for each comparison, are displayed in Supplementary Figure S2.

### 3.2 Extensions of PCA-based ROI selection beyond functional connectivity

#### 3.2.1 Group differences in structural connectivity

**Fig. 5.**
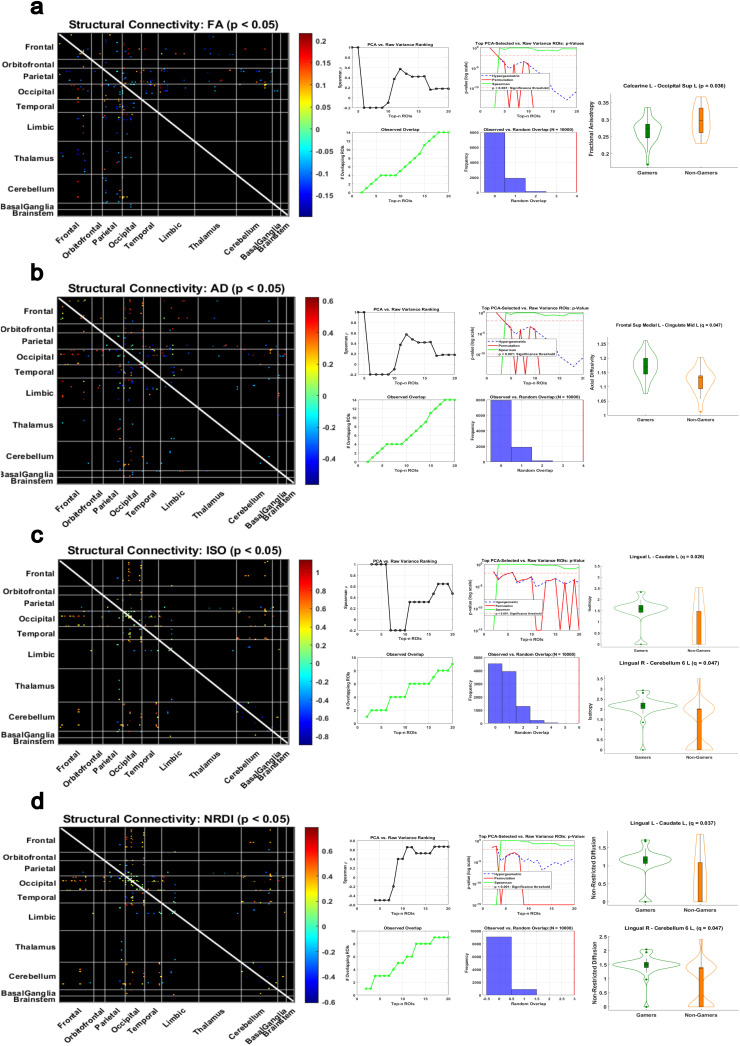
Structural Connectivity Differences Filtered by Top rcPCA ROIs. Group-level differences in structural connectivity (SC) between gamers and non-gamers using PCA-based ROI selection across four microstructural diffusion metrics. Each panel shows a full SC matrix of significant group differences (*p* < 0.05), validation of PCA-based selection stability, and violin plots of the strongest observed effects (*p* < 0.05). In the violin plots, gamers are shown in green and non-gamers are shown in orange. PCA-based filtering revealed consistent and interpretable differences across SC measures. All effects shown reflect comparisons involving top PCA-selected ROIs. (a) Fractional anisotropy (FA): a significant difference was observed in the connection between Calcarine L and Occipital Sup L, which was also associated with slower response times in behavioral analysis. (b) Axial diffusivity (AD): Group differences were observed in connections involving Frontal Sup Medial L and Cingulate Mid L. (c) Isotropic volume fraction (ISO): effects were found in Lingual L—Cerebellum 6 L and Lingual R—Cerebellum 6 L. (d) Non-restricted diffusion imaging (NRDI): the same cerebellar projections from Lingual L and Lingual R remained significant.

Structural connectivity (SC) analysis using diffusion measures, fractional anisotropy (FA), axial diffusivity (AD), isotropy (ISO), and non-restricted diffusion imaging (NDRI) revealed significant group-level differences between gamers and non-gamers involving rcPCA-derived ROIs (*p* < 0.05), as shown in Figure 5. For FA, reduced connectivity in non-gamers was observed between the left calcarine cortex and the left superior occipital gyrus (*p* = 0.036, *d* = –0.78), a connection also associated with slower response times in behavioral analysis (see Fig. 7).

In the AD measure, a robust group difference emerged between the left superior medial frontal gyrus and the left middle cingulate cortex (*p* = 0.0003, *q* = 0.047, *d* = 1.16), the only FA or AD comparison to survive FDR correction. ISO-based analyses revealed stronger connectivity in gamers between the left lingual gyrus and the left caudate (*p* = 0.0002, *q* = 0.026, *d* = 1.22) and between the right lingual gyrus and the left cerebellar lobule 6 (*p* = 0.0007, *q* = 0.047, *d* = 0.96). These region pairs also showed significant differences in the NDRI measure, with both comparisons maintaining significance following FDR correction.

Absolute effect sizes across all measures were consistently large, with *d* values ranging from 0.78 to 1.22. Full statistical results, including all *p*-values, FDR-adjusted *q*-values, and Cohen’s *d* effect sizes for each comparison, are reported in Supplementary Figure S2.

#### 3.2.2 Structure–function coupling

**Fig. 6.**
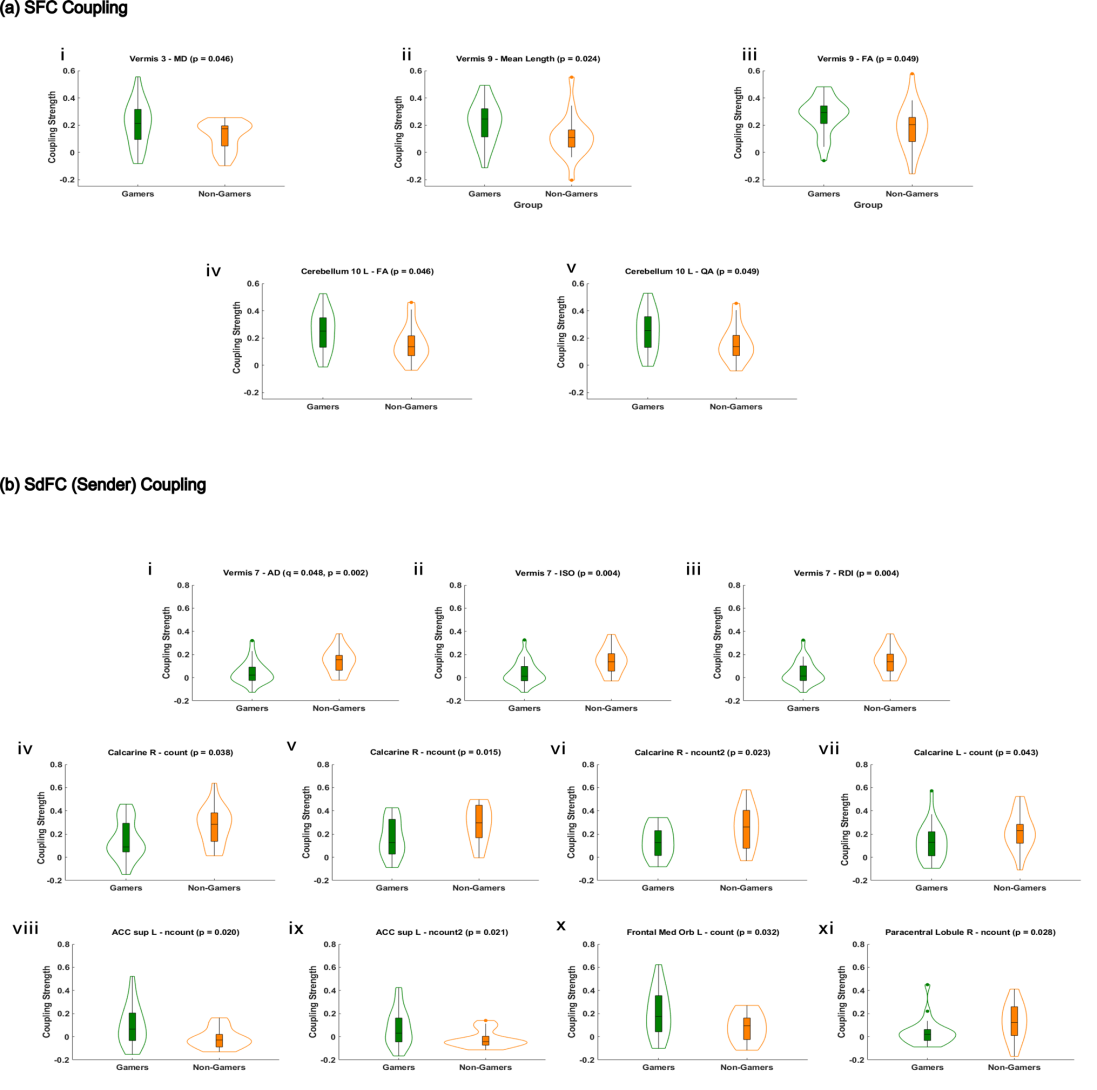
Group-level differences in structure-function coupling involving top rcPCA ROIs. Group-level differences in structure-function coupling between gamers and non-gamers using PCA-based ROI selection. Panels within each section are ordered by appearance in the text: SFC (a) i-v and SdFC (sender) (b) i-xi. In the violin plots, gamers are shown in green and non-gamers in orange. (a) SFC coupling: Significant group differences (p < 0.05) were observed across several structural connectivity measures, including mean diffusivity (MD), mean length, fractional anisotropy (FA), and quantitative anisotropy (QA), in connections involving Vermis 3, Vermis 9, and Cerebellum 10 L. (b) SdFC coupling (sender): Group differences in sender-based structure-function coupling strength (p < 0.05) were observed, with significant effects in Vermis 7 across intensive measures such as axial diffusivity (AD), isotropy (ISO), and restricted diffusion imaging (RDI). Additional effects were observed in the Calcarine cortex (R and L), ACC sup L, Frontal Med Orb L, and Paracentral Lobule R when coupled with extensive measures including count, ncount, and ncount2. All effects reflect comparisons involving top PCA-selected ROIs. Sender-based SdFC coupling was selected to parallel SFC coupling based on similar FC and dFC PCA validation profiles prior to coupling. Validation of PCA-based ROI selection followed the same procedure as in previous analyses of FC, dFC, and SC connectivity.

Structure–function coupling measures the alignment between a region’s structural connectivity measure and its corresponding capacity for functional load (Fotiadis et al., 2024). To quantify this relationship, Pearson correlations were computed between structural connectivity (SC) and both functional connectivity (FC) and directed functional connectivity (dFC sender mode). The undirected correlation with FC is referred to as SFC, while the correlation with sender-mode dFC is termed SdFC (sender). As shown in Figure 6a, gamers exhibited significantly stronger coupling in the cerebellum. These included Vermis 3 with mean diffusivity (*p* = 0.046, *d* = 0.64), Vermis 9 with both mean length (*p* = 0.024, *d* = 0.64) and fractional anisotropy (*p* = 0.049, *d* = 0.55), and Cerebellum 10 L with fractional anisotropy (*p* = 0.046, *d* = 0.65) and quantitative anisotropy (*p* = 0.049, *d* = 0.65).

In the SdFC sender condition shown in Figure 6b, non-gamers showed significantly stronger coupling in Vermis 7 across multiple measures, including axial diffusivity (*p* = 0.002, *q* = 0.048, *d* = –1.00), isotropy (*p* = 0.004, *d* = –0.97), and restricted diffusion (*p* = 0.004, *d* = –0.97). Additional effects favoring non-gamers were found in the right calcarine cortex for count (*p* = 0.038, *d* = –0.74), normalized count (ncount;*p* = 0.015, *d* = –0.82), and inverse-length weighted normalized count (ncount2;*p* = 0.023, *d* = –0.82), as well as in the left calcarine cortex (*p* = 0.043, *d* = –0.59). Gamers, in contrast, exhibited stronger SdFC sender coupling in the left supracallosal anterior cingulate cortex for both ncount (*p* = 0.020, *d* = 0.82) and ncount2 (*p* = 0.021, *d* = 0.83), in the left medial orbitofrontal cortex for count (*p* = 0.032, *d* = 0.74), and in the right paracentral lobule for ncount (*p* = 0.028, *d* = –0.67).

Effect size magnitudes were moderate to large across both coupling modes, with *d* values ranging from 0.55 to 1.00. The Vermis 7–axial diffusivity pair was the only comparison to survive FDR correction. These findings highlight distinct structure–function coupling profiles between groups, particularly in cerebellar and occipital regions. Full statistical results, including all *p*-values, FDR-adjusted *q*-values, and Cohen’s *d* effect sizes for each comparison, are reported in Supplementary Figure S2.

### 3.3 Brain–behavior relationships

**Fig. 7.**
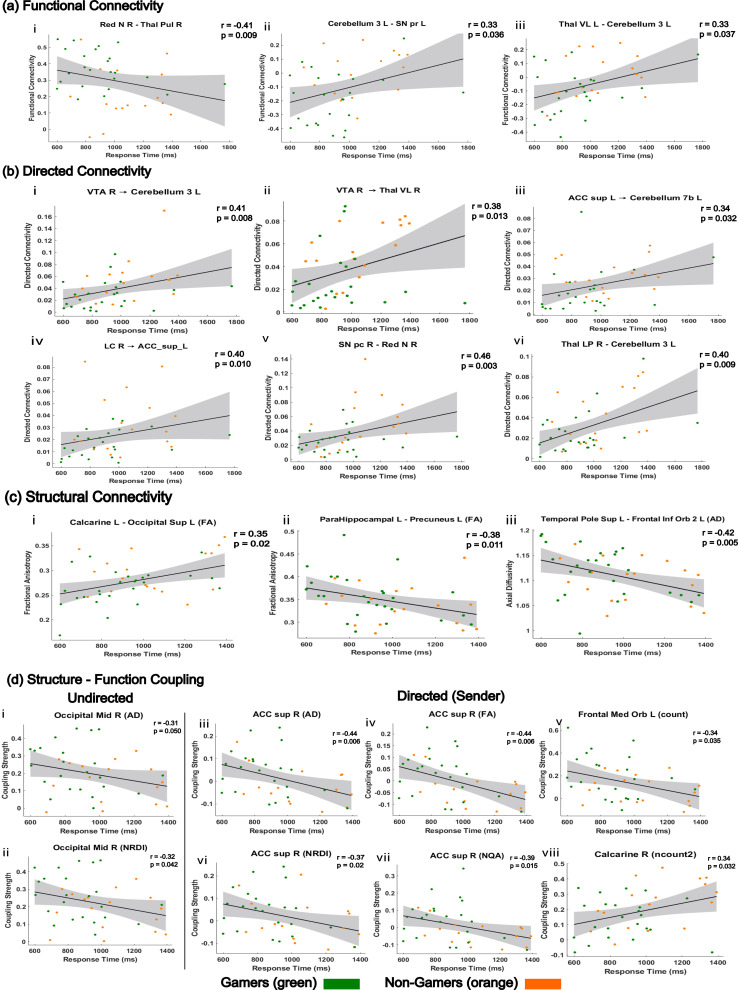
Behaviorally Relevant Connectivity and Coupling Involving Top PCA ROIs. Brain-behavior relationships linking connectivity and coupling strength to response time across cohorts via Spearman’s rank correlation (ρ). Positive correlations indicate slower responses, and negative correlations indicate faster responses. Gamers are shown as green dots and non-gamers as orange dots. (a) Functional connectivity (FC): Faster response times were associated with stronger FC between the red nucleus and thalamus (PuL R). In contrast, slower responses were associated with stronger FC between midbrain (SN pr L) and thalamic (Thal VL L) regions connected to the cerebellum. Sublabels (i-iii) are ordered from lowest to highest p-value. (b) Directed connectivity (dFC): Slower responses were linked to increased directed influence among midbrain (VTA R, SN pc R), thalamic (Thal VL R), cerebellar, and anterior cingulate (ACC sup L) pathways. Sublabels are grouped by dFC modality, sender (i-iii), receiver (iv), and total (v-vi), and are ordered from lowest to highest p-value within each modality. (c) Structural connectivity (SC): FA- and AD-based measures showed significant associations with behavior. Higher FA between Calcarine L and Occipital Sup L predicted slower performance. Sublabels (i-iii) are grouped by SC measure, FA (i-ii) and AD (iii), following the order of presentation in the Results. (d) Structure-function coupling: Both SFC and sender-based SdFC coupling strength tracked with response time. Sublabels are grouped by coupling type, SFC (i-ii) and sender-based SdFC (iii-viii). Faster responses were associated with stronger coupling in frontal, anterior cingulate, and mid-occipital regions.

Response time was significantly associated with connectivity strength and structure–function coupling across modalities. Here and in Figure 7, *r* denotes Spearman’s rank correlation (ρ) between RT and connectivity. Because smaller RT means faster performance, negative *r* (downward slope) tracks with faster responses, whereas positive *r* tracks with slower responses.

In functional connectivity, faster responses were linked to stronger connectivity between the right red nucleus and the right pulvinar thalamus (*r* = –0.41, *p* = 0.009), whereas slower responses were associated with increased connectivity in cerebellar and thalamic circuits, including the left cerebellar lobule 3 and the right substantia nigra pars compacta (*r* = 0.33, *p* = 0.036), as well as the left ventrolateral thalamus and the left cerebellar lobule 3 (*r* = 0.33, *p* = 0.037).

In directed connectivity, slower responses were correlated with increased influence from the right ventral tegmental area to the left cerebellar lobule 3 (*r* = 0.41, *p* = 0.008), from the right ventral tegmental area to the right ventral lateral thalamus (*r* = 0.38, *p* = 0.013) from the right substantia nigra pars compacta to the right red nucleus (*r* = 0.46, *p* = 0.003), and from the left supracallosal anterior cingulate cortex to the left cerebellar lobule 7b (*r* = 0.34, *p* = 0.032). Additional significant effects included connections from the right lateral posterior thalamus to the left cerebellar lobule 3 (*r* = 0.40, *p* = 0.009) and from the right locus coeruleus to the left supracallosal anterior cingulate cortex (*r* = 0.40, *p* = 0.010).

In structural connectivity, slower responses were associated with higher fractional anisotropy between the left calcarine cortex and the left superior occipital gyrus (*r* = 0.35, *p* = 0.020), while faster responses were linked to higher fractional anisotropy between the left parahippocampal gyrus and the left precuneus (*r* = –0.38, *p* = 0.011), and to higher axial diffusivity between the left superior temporal pole and the left orbital part of the inferior frontal gyrus (*r* = –0.42, *p* = 0.005).

In structure–function coupling, faster response times were associated with stronger coupling in the right mid-occipital cortex, including axial diffusivity (*r* = –0.31, *p* = 0.050) and non-restricted diffusion imaging (*r* = –0.32, *p* = 0.042). Stronger coupling in the right supracallosal anterior cingulate cortex was also associated with faster responses across multiple measures: axial diffusivity (*r* = –0.44, *p* = 0.006), fractional anisotropy (*r* = –0.44, *p* = 0.006), normalized quantitative anisotropy *(r* = –0.39, *p* = 0.015), and non-restricted diffusion imaging (r = –0.37, *p* = 0.020). In sender-mode coupling, faster responses were further linked to greater structure–function alignment from the right anterior cingulate cortex (*e.g.,* FA: *r* = –0.44, *p* = 0.006; NRDI: *r* = –0.37, *p* = 0.020; NQA: *r* = –0.39, *p* = 0.015), as well as from the left medial orbital frontal cortex (*r* = –0.34, *p* = 0.035) and the left calcarine cortex (*r* = –0.34, *p* = 0.032). All brain–behavior correlations supporting these effects are displayed in Figure 7.

## 4 Discussion

### 4.1 A novel PCA-based framework for region selection

To address the challenges of navigating the complexity of whole-brain connectivity matrices, we developed a novel PCA-based framework that reimagines the role of principal component analysis (PCA) in neuroimaging. Rather than relying on anatomical constraints or arbitrary statistical cutoffs, this method decomposes subject-by-connection matrices into orthogonal principal components and computes the eigenvalue-weighted sum of absolute contributions for the top user-defined regions across all components. The result is a global variance contribution score for each brain region, allowing us to prioritize the most informative regions for downstream group comparisons based on their contribution to explained variance. For our analysis, we selected the top 20 regions across all components, up to an explained variance threshold of 80%, to emphasize the most relevant contributors for downstream analysis while controlling for stability.

Crucially, this region-cumulative PCA method (rcPCA) leverages the mathematical structure of PCA (Mwangi et al., 2014; Wall et al., 2002) to form an orthonormal basis over the space of brain regions, enabling a principled and interpretable ranking of ROI importance. By weighting regional contributions according to the variance explained by each principal component, the method prioritizes dominant, structured inter-subject variance. This orthogonal decomposition avoids redundancy, permitting straightforward accumulation of variance-weighted contributions.

Moreover, rcPCA flexibly handles both undirected and directed connectivity matrices. For directed measures, such as Granger causality, the method separately calculates contribution scores for the sender, receiver, and total contributions, allowing for a nuanced interpretation of directional asymmetries in brain networks. This increases the interpretability and relevance of the selected ROIs. Altogether, this novel application of PCA provides a mathematically robust alternative to traditional thresholding and anatomical filtering, focusing on variance-based selection that generalizes across structural, functional, directed connectivity, and coupling modalities.

We developed a multi-pronged validation strategy to evaluate the internal consistency and robustness of rcPCA using rank correlation, permutation testing, and hypergeometric overlap statistics with a conservative significance threshold of *p* < 0.001 as our primary validation criteria. This strategy enabled a drastic reduction in the number of statistical comparisons required for downstream testing, significantly improving our ability to detect FDR-corrected (*q* < 0.05) results that would have been obscured by multiple comparison corrections.

In our previous study, we applied a structurally constrained functional neuroimaging analysis, limiting connectivity assessments to anatomically plausible tracts informed by white matter tractography (Cahill & Dhamala, 2025). While this approach enhanced biological plausibility, it was inherently limited by tractography reconstruction assumptions and macro-level user-defined parameters such as allowable streamline length and angular thresholds. The rcPCA method offers a complementary perspective by identifying dominant, behaviorally relevant patterns of variance without relying on anatomical priors. Unlike anatomical filtering, which can miss regions not tightly bound to known white matter pathways, PCA captures emergent structure across subjects and modalities. By combining tractography-informed constraints to ensure biological plausibility with rcPCA to filter for the most robust patterns in inter-subject variability, we constructed a methodological framework capable of supporting a comprehensive and principled investigation of the neuroplastic adaptations associated with action video game experience.

To summarize, rcPCA ranks ROIs via variance-weighted PC loadings (80% cumulative variance), keeps the top 20 per modality (including dFC sender/receiver/total), and reduces multiple comparisons to a stable, parcellation-agnostic shortlist for group-level and brain–behavior analyses.

### 4.2 Group differences between gamers and non-gamers across modalities

Group-level comparisons between gamers and non-gamers revealed widespread differences across multiple connectivity domains, all of which involved at least one PCA-identified region of interest (ROI).

In functional connectivity (FC), shown in Figure 3, gamers exhibited significantly stronger FC between the right red nucleus and the right pulvinar thalamus, the left cerebellum lobule 3 and the left caudate nucleus, and the left cerebellum lobule 3 and the right superior temporal pole. In contrast, non-gamers showed stronger FC between the left cerebellum lobule 3 and the left intralaminar thalamus, the left cerebellum lobule 3 and the left substantia nigra pars reticulata, and the left cerebellum lobule 3 and the ventrolateral thalamus bilaterally. All FC differences, except for the connections involving the ventrolateral thalamus, survived FDR correction. Several of these FC connections were also significantly correlated with response time (RT), indicating behavioral relevance. These patterns suggest that non-gamers may rely more heavily on feedback-based loops involving cerebellar–thalamic–midbrain circuits, potentially as a mechanism to reduce uncertainty during motor action selection. Effect sizes were consistently large, with Cohen’s *d* magnitudes ranging from 0.76 to 1.26.

In directed functional connectivity (dFC), presented in Figure 4, sender-mode results revealed a robust group difference in directed influence favoring non-gamers from the left superior anterior cingulate cortex to the right ventrolateral thalamus, which survived FDR correction. Additional uncorrected differences favoring non-gamers included projections from the left superior anterior cingulate cortex to the left cerebellum lobule 7b, from the right ventral tegmental area to the right ventrolateral thalamus, and from the right ventral tegmental area to the left cerebellum lobule 3. Notably, each of these connections also showed significant positive correlations with RT.

Receiver-mode differences also favored non-gamers, with several directed connections showing stronger influence toward key target regions. These included projections from the left cerebellum lobule 3 to the left superior anterior cingulate cortex, from the right ventral tegmental area to the left cerebellum lobule 3, and from the right locus coeruleus to the left superior anterior cingulate cortex. Several of these connections also showed significant positive correlations with RT. Total-mode results closely mirrored the sender- and receiver-mode patterns, with notable unique group differences such as increased connectivity from the right red nucleus to the right substantia nigra pars compacta and from the left thalamus to the left cerebellum lobule 3 in non-gamers.

The consistency of these effects across all directed functional connectivity (dFC) modes, coupled with moderate to large effect sizes (*d* = 0.59 to 1.00), reinforces the earlier findings from tractography-constrained analyses (Cahill & Dhamala, 2025), which revealed that non-gamers exhibit a greater number of directed connections. The current results build on that observation, suggesting that non-gamers engage in a broader, more distributed pattern of targeted information flow, likely reflecting compensatory network recruitment to signal the need for an imminent motor response during rapid visuomotor decision making.

In structural connectivity (SC) analysis using diffusion measures (FA, AD, ISO, and RDI), we observed group-level differences involving PCA-derived regions of interest (*p* < 0.05) as shown in Figure 5. For FA, a notable and seemingly counterintuitive difference emerged that FA was significantly higher in non-gamers between the left calcarine cortex and the left superior occipital gyrus—early visual areas (Huff et al., 2025), which also had a positive correlation with RT thus associated with slower response times in behavioral analysis (see Fig. 7). This finding suggests that non-gamers may over rely on early stage visual processing to compensate for inefficiencies further downstream. For AD, group differences emerged between the left superior medial frontal gyrus and the left mid-cingulate cortex (*q* < 0.05). ISO revealed significant effects in connections between the right lingual gyrus and the left cerebellum lobule 6, as well as between the left lingual gyrus and the left caudate nucleus. Both connections remained significant in NRDI and under FDR correction. Absolute effect sizes across all SC measures were substantial, with Cohen’s *d* magnitudes ranging from 0.78 to 1.22, indicating consistently large group-level differences.

Structure–function coupling findings displayed in Figure 6 provided further insight into group differences by measuring the alignment between a region’s structural connectivity profile and its capacity for functional load (Fotiadis et al., 2024). Gamers exhibited stronger structure–function coupling (SFC) within cerebellar regions, including vermis lobule 3 (MD), vermis lobule 9 (mean length, FA), and the left cerebellum lobule 10 (FA, QA). In contrast, non-gamers showed stronger structure-directed functional coupling (SdFC sender) in bilateral calcarine cortices and vermis lobule 7. One of these connections, involving vermis lobule 7 and AD-based SdFC sender coupling, survived FDR correction.

Gamers also demonstrated stronger SdFC sender coupling in key frontal and cingulate regions, including the left superior anterior cingulate cortex (ncount, ncount2), the left medial orbital frontal cortex (count), and the left paracentral lobule (ncount). These findings underscore a functional distinction between undirected synchrony, as measured by SFC, and directional signaling capacity, as captured by SdFC sender. Stronger coupling in gamers may reflect more efficient transmission channels for dynamic signaling, consistent with prior evidence of increased dorsal attention to salience network (DAN-to-SN) switching, and which the superior anterior cingulate cortex is a core node in the salience network (SN) (Jordan & Dhamala, 2022a).

Across modalities, gamers exhibit a feedforward, salience-driven profile. Non-gamers rely more on cerebellar, thalamic, and midbrain feedback and early visual scaffolding, and show dFC edges that increase with RT, indicating slower responses.

### 4.3 Brain–behavior relationships

Connectivity and coupling strengths across all modalities were significantly associated with response time (RT) (Fig. 7). These associations reinforce the behavioral relevance of group differences and suggest that the specific network configurations observed in gamers versus non-gamers map divergent visuomotor strategies. Non-gamers exhibited more engagement in regions and connections involving cerebellar–midbrain–thalamic circuits, which were consistently associated with slower responses and may reflect a less efficient allocation of cognitive resources.

#### 4.3.1 Functional connectivity (FC)

Faster RTs, indicated by negative correlations, were linked to stronger FC between the red nucleus and the pulvinar thalamus, a connection that is canonically linked to limb control. In contrast, slower RTs were associated with stronger FC in cerebellar–thalamic/cerebellar–midbrain, connections particularly between cerebellum 3 L and thalamus intralaminar L, substantia nigra pars reticulata L, and thalamus ventrolateral L/R—all of which favored non-gamers. These patterns suggest that overreliance on cerebellar–thalamic error correction signals (Ide & Li, 2011), which would slow down visuomotor transformation, promote tighter inhibitory control, evidenced by increased substantia nigra–thalamus ventrolateral synchrony in non-gamers (Walter & Shaikh, 2014). This elevated substantia nigra–thalamus ventrolateral synchrony suggests reduced motor readiness, consistent with increased inhibitory gating of thalamic output, and reflects a diminished capacity to swiftly resolve competing motor plans (Sonne et al., 2025), ultimately delaying commitment to action selection under uncertainty and prolonging response time.

Overall, FC results suggest gamers show greater motor readiness, reflected in stronger red nucleus–pulvinar thalamus connectivity linked to faster RTs, whereas non-gamers rely more on cerebellar–thalamic and cerebellar–midbrain connections associated with slower RTs.

#### 4.3.2 Directed functional connectivity (dFC)

All three sender-mode connections that favored non-gamers—anterior cingulate cortex superior L → cerebellum 7b L, ventral tegmental area R → thalamus ventrolateral R, and ventral tegmental area R → cerebellum 3 L—also showed significant positive correlations with RT, indicating slower responses. Likewise, several receiver-mode connections that favored non-gamers (e.g., cerebellum 3 L → anterior cingulate cortex superior L, ventral tegmental area R → cerebellum 3 L, locus coeruleus R → anterior cingulate cortex superior L) were also positively correlated with RT. These results point to a more distributed routing of information in non-gamers associated with cerebellar error correction (Caligiore et al., 2017), stress response (Borodovitsyna et al., 2020) (locus coeruleus), and dopaminergic reward-seeking impulsiveness (Aurelian et al., 2016) (ventral tegmental area) VTA. In this context, this increased signaling from VTA may be acting as a compensatory mechanism for a lack of goal-directed strategy, one incurs a performance cost.

Putting this together, our dFC results indicate that non-gamers rely more on feedback-oriented cerebellar–thalamic–midbrain pathways whose influence scales positively with RT (*i.e*., slower responses).

#### 4.3.3 Structural connectivity (SC)

Regarding structural connections, the FA connection between the calcarine cortex and superior occipital cortex predicted slower RTs in non-gamers. While gamers exhibited elevated FA along the left superior occipital–inferior parietal dorsal stream (Cahill et al., 2024), neither this tract nor the part of the dorsal stream between the calcarine–occipital FA connection showed a direct behavioral relationship. In contrast, FC between the left superior occipital gyrus and superior parietal lobule was significantly associated with faster RTs. Moreover, SC-constrained analyses revealed that higher local efficiency in the left superior occipital cortex predicted faster responses, whereas greater node degree was associated with slower performance (Cahill & Dhamala, 2025). Additionally, drawing from our SdFC (sender) coupling results, the coupling of the number of tracts with the calcarine sulcus was associated with slower response times and was elevated in non-gamers.

Together, these findings suggest that non-gamers rely more heavily on investing cognitive resources into early visual processing, likely putting more effort into object discrimination and resolution of individual dot trajectories during rapid visuomotor decisions to compensate for reduced visuomotor integration. Since the dorsal stream’s structural integrity was previously shown not to be predictive of performance in prior work within the same dataset (Cahill et al., 2024), the tracts contributing to slower RTs are more likely to occur outside of this core visuomotor pathway (Goodale & Milner, 1992; Kravitz et al., 2011; Mishkin et al., 1983). This reinforces the interpretation that while early sensory processing is essential, behavioral efficiency is more tightly linked to visuomotor transformation and motor-readied, goal-driven action selection. Cognitive resources appear to yield greater behavioral returns when invested in converting sensory input into action once a satisfactory perceptual benchmark is reached, rather than in the monotonic refinement of early-stage encoding, which exhibits diminishing returns.

Additional behaviorally relevant structural connections reinforce this interpretation. Faster responses were associated with higher axial diffusivity (AD) between the superior temporal pole and inferior frontal orbital cortex regions critical for integrating high-value sensory information (Herlin et al., 2021) with goal-directed evaluation (Rolls, 2023; Rudebeck & Rich, 2018; Shi et al., 2023). In this context, the superior temporal pole likely receives scene-specific contextual input from the parahippocampus, enabling the inferior frontal orbital cortex to transform the learned value of this contextual information into action during visuomotor decisions.

Similarly, greater fractional anisotropy (FA) between the parahippocampus and left precuneus was linked to faster RTs. The parahippocampus plays a key role in recognizing scene-specific spatial relationships (Burgess & O’Keefe, 2003), while the precuneus supports visuospatial imagery and internal simulation (Blihar et al., 2020; Cavanna & Trimble, 2006; Hahn et al., 2006)—both directly relevant to anticipating movement trajectories based on relative motion cues.

Taken together, SC findings indicate an early visual bias in non-gamers linked to slower RTs: higher FA in the calcarine–superior occipital connection predicted slower responses. By contrast, gamer-linked tracts support integration of higher-level information and are associated with faster RTs, exemplified by higher AD between the superior temporal pole and inferior frontal orbital cortex—regions implicated in value-based sensory integration and goal-directed evaluation.

#### 4.3.4 Structure–function coupling

Structure–function coupling metrics provided further insight into behaviorally relevant dynamics measuring how well a region’s anatomical infrastructure aligns with its functional load (Fotiadis et al., 2024). Both intensive (e.g., FA, AD, NQA, NRDI) and extensive (e.g., count, ncount2) structural metrics were shown to be behaviorally relevant.

In this study, individuals with stronger NDRI–FC and AD–FC coupling in the right mid-occipital region exhibited faster response times. Given this region’s established role in spatial information processing (Renier et al., 2010), this behaviorally relevant finding suggests that stronger coupling may reflect greater alignment between incoming visual load and the region’s capacity to relay spatial information to downstream nodes, thereby facilitating response selection. Prior work from our SC-constrained analyses showed that gamers exhibit greater local efficiency in both SC-constrained FC and SC-constrained dFC networks involving this same region (Cahill & Dhamala, 2025). This suggests that, at equivalent levels of coupling strength, gamers’ networks are more structurally optimized to disseminate spatial information, enabling faster integration with behaviorally relevant areas. Such an architecture may indirectly support more efficient visuomotor transformations, even when the degree of structure–function alignment is comparable between groups.

The coupling of count with sender dFC in the left frontal medial orbital cortex was greater in gamers and negatively correlated with response time, indicating faster behavioral performance. This aligns with earlier findings showing that faster RTs were also facilitated by greater axial diffusivity between the left superior temporal pole and the left inferior frontal orbital cortex. Both medial and inferior frontal orbital regions are implicated in integrating value-based information with action planning (Rolls, 2023; Rudebeck & Rich, 2018). In this context, this bolsters previous findings that gamers engage in more feedforward, top–down selection of goal-relevant motor responses based on high-level contextual information such as relative motion cues extracted from the scene, including the motion of target dots relative to distractors (Cahill & Dhamala, 2025). The right superior anterior cingulate results were consistent with prior evidence of greater DAN→SN network switching in gamers and the role of superior ACC as a core node in the salience network, SN (Jordan & Dhamala, 2022a). Additionally, as discussed earlier, increased reliance on early visual processing in non-gamers may explain the higher ncount2 coupling with sender dFC in the calcarine R. This finding supports our claim that non-gamers are overly dependent on early stage visual input, which likely contributes to downstream bottlenecks during visuomotor transformation and action execution—ultimately slowing visuomotor decision-making response times compared with gamers.

In sum, our structure–function coupling results suggest that gamers exhibit stronger coupling in regions supporting salience, learned value, and action planning. This pattern is associated with faster RTs, whereas non-gamers show stronger early visual coupling linked to slower RTs.

### 4.4 Synthesis of findings across modalities

Across modalities, a consistent pattern emerged that faster response times were associated with stronger connectivity in circuits involved in transforming high-value sensory information into goal-directed action and supporting goal-directed motor execution, that is*,* regions implicated in resolving perceptual ambiguity more effectively tracked with faster RT. Central to this process was the superior anterior cingulate cortex (ACC sup), a region traditionally associated with conflict monitoring and the resolution of competing motor plans (Brockett & Roesch, 2021). More recent work suggests the ACC functions as a key hub for uncertainty-driven cognitive control, particularly in dynamic and feedback-sensitive decision environments (Chen et al., 2024; Monosov, 2017; Monosov et al., 2020; Mushtaq et al., 2011). These areas likely serve to clarify salience signals and commit to goal-relevant actions once monitoring demands have been satisfied. The superior ACC was previously found to be a significant node involved with the increased DAN-to-SN interaction observed in gamers, which tracked with improved RT (Jordan & Dhamala, 2022a) and within the right ACC, a node by which enhanced SdFC sender coupling was tracked with improved response times across multiple SC measures.

Conversely, increased activity among regions involved in motor correction, stress reactivity, and dopaminergic signaling was positively correlated with response time, indicating slower performance and stronger involvement in non-gamers. This pattern may reflect reduced access to high-value salient cues, as suggested by greater engagement of early visual areas and cerebellar–midbrain–thalamic circuits (Caligiore et al., 2017; Popa & Ebner, 2019; Seidler et al., 2013). These compensatory pathways likely reflect increased reliance on bottom–up processing and corrective feedback, with the ACC recruited to manage elevated uncertainty during response selection.

Examples of regions implicated in resolving perceptual ambiguity included the left medial frontal orbital cortex, an executive region critical for integrating value-based information with motor planning. SdFC sender coupling with streamline count in this region was significantly stronger in gamers (*p* = 0.032, *d* = 0.74) and negatively correlated with response time (*r* = –0.34, *p* = 0.035), which tracks with better performance. Another example includes increased synchrony, measured by functional connectivity, between the red nucleus and the pulvinar thalamus, reflecting enhanced bottom–up and top–down coordination. This connection likely contributes to perceptual disambiguation by signaling motor readiness (Basile et al., 2021; Brockett et al., 2020; Krimmel et al., 2025), effectively facilitating the “I’m ready to press the button” moment.

This red nucleus and pulvinar thalamus finding parallels prior work in Go/No-Go paradigms, where red nucleus activity modulates motor output based on recent trial history, speeding responses after Go trials, and promoting caution after Stop trials. Critically, red nucleus neurons amplify directional signals during successful Stop trials, suggesting a role in reshaping ongoing motor plans when initial responses must be inhibited. This capacity for rapid, context-sensitive motor adjustment reflects a form of feedforward control, in which the system anticipates task demands and dynamically tunes motor output in real time. The functional connection between the red nucleus and pulvinar thalamus is supported by a group-level difference favoring gamers (*p* = 0.0061, *q* = 0.02, *d* = 0.85), and negatively correlated with response time (*r* = –0.41, *p* = 0.009), which suggests that long-term exposure to high-stakes, fast-paced environments fosters a neurocognitive strategy that prioritizes feedforward conflict resolution—facilitating swift action and flexible, real-time motor adjustments under uncertainty.

Our results suggest that gamers exhibit a more feedforward, proactive strategy for visuomotor transformation, and a more optimized neural architecture for fast, adaptive, goal-directed behavior—one that more readily anticipates possible task outcomes and enables flexible, real-time motor corrections. By reducing internal uncertainty more efficiently, this yields a more effective cognitive architecture for action selection under dynamic, time-sensitive conditions. Overall, gamers demonstrate enhanced top–down cognitive clarity, unobstructed translation of learned value into action, and bottom–up motor readiness when making visuomotor decisions under uncertainty. This configuration reduces the need for prolonged internal conflict resolution between competing motor plans and offers what may be a functionally parsimonious explanation for their accelerated decision making compared with non-gamers.

In the context of our modified moving-dots task, this strategy was expressed as greater reliance on top–down selection of goal-directed responses based on scene-specific contextual cues, such as the motion of target dots relative to distractors. Gamers adapted more effectively to either possible outcome of a 50/50 visuomotor decision, reflecting enhanced cognitive flexibility and attentional control.

Taken together, our results support the hypothesis that long-term action video games (AVG) play reflects neuroplastic refinements that align with greater feedforward processing, motor readiness, improved anticipation, integration, and transformation of high-value visual cues into goal-directed action in gamers, which is expected to reduce visuomotor surprise by shifting toward superior decision-making strategies—ones that more effectively resolve prediction error by minimizing internal conflict regarding competing motor plans. This facilitates a more optimal cognitive state for visuomotor decisions, one that is primed to rapidly incorporate salient, task-relevant information and execute swift, accurate, and decisive actions.

These reflected neuroplastic refinements observed in gamers are consistent with a reallocation of cognitive resources toward circuits that minimize visuomotor surprise more efficiently. Cognitive Resource Reallocation (CRR), therefore, provides a plausible mechanistic explanation of how repeated engagement with strenuous visuomotor demands may gradually refine neural configuration over time. This shift toward optimizing internal conflict resolution could potentially indicate a broader principle regarding how cognitive systems respond to prolonged cycles of strenuous task engagement and recovery—gradually reallocating resources to stabilize changes in cognitive action over time. These changes may promote local refinements that enhance overall efficiency during task performance by targeting regions and connections most involved in reducing task-induced strain.

Within the context of AVG experience, this would lead to neuroplastic refinements that translate into more efficient visuomotor decision making—a common and frequent demand during gameplay, where errors often come with significant costs. Over time, the brain learns to prioritize high-value visual cues, promote goal-directed action, and increase motor readiness to respond to uncertainty in dynamically changing environments. This ultimately would result in quantifiable improvements, establishing a new set point by which the brain makes visuomotor decisions, leading to the ~190 ms response time advantage observed in gamers, without any loss in accuracy.

As gamers repeatedly engage in high-stakes, rapid-response tasks, the CRR framework posits that the brain reallocates resources toward pathways that facilitate more proficient AVG performance. These reallocations map cleanly onto cognitive improvements consistently reported in the literature, including enhanced visual acuity (Green & Bavelier, 2007), visuomotor integration (Cahill et al., 2024; Granek et al., 2010), attentional control (Bavelier & Green, 2019), and cognitive flexibility (Glass et al., 2013). The point to be emphasized here is that, rather than endlessly refining early sensory representations beyond a sufficiently reliable threshold, our findings suggest that long-term AVG experience reflects refinements that encourage the brain to avoid diminishing returns, given that the priority is to execute the task of playing an AVG as efficiently as possible. Over repeated cycles of task engagement and recovery, resources are reallocated toward downstream processes more directly involved in effective gameplay, particularly those supporting visuomotor decision making and perception–action coupling. This shift offers a comprehensive and mechanistically grounded account of the functional gains observed in gamers and provides a clear neural signature of long-term adaptive plasticity associated with AVG play.

### 4.5 Methodological strengths, limitations, and future directions

PCA is fully data-driven and agnostic to anatomical priors, enabling it to reveal latent patterns of intersubject variability that might be missed in anatomically constrained pipelines. The rcPCA ROI selection method presented here offers several practical strengths. By identifying relevant ROIs *a priori* based on structured variance, the approach reduces the multiple comparisons burden inherent in full-brain ROI-wise testing. Rather than applying statistical correction across tens of thousands of univariate tests, corrections are reserved for only the most informative regions and their corresponding connections. This not only enhances statistical power but also improves interpretability and replicability, particularly in studies with modest sample sizes.

Our study recruited a sample of healthy young adults, allowing us to isolate the effects of long-term video game playing while minimizing potential confounds. However, this design choice also imposes certain limitations. First, the dataset in this study captures a cross-sectional snapshot of individuals with long-term action video game experience. As such, it does not permit inferences about the rate of neuroplastic adaptation over time, limiting the ability to make direct causal claims. Determining how these changes evolve would require longitudinal studies and clinical training interventions.

Participants were recruited from university campuses with presumably similar educational backgrounds; however, we did not explicitly screen for education levels or cognitive ability, meaning we cannot establish direct correlations between baseline cognitive performance and task outcomes. Additionally, our sex distribution was not balanced between gamers and non-gamers, limiting our ability to examine sex-specific differences in brain and behavioral responses.

A small but consistent male advantage in vision-based response times and visual–spatial working memory has been reported across the literature (Mathew et al., 2020; Schrauwen et al., 2014; Voyer et al., 2017). Specifically, recent work examining sex differences in visuomotor tracking and response times suggests an average sex difference of approximately 20 ms favoring males (Mathew et al., 2020). This magnitude is substantially smaller than the ~190 ms (*p* = 2.05 × 10^−70^) response time difference observed in gamers in this dataset (Jordan & Dhamala, 2022b) and is, therefore, unlikely to be explained by sex alone. Females have also been observed to have a more proactive visuomotor decision strategy than males, yielding a slight advantage in accuracy but slower responses due to the enhanced visual-to-motor processing observed in males (Bianco et al., 2020). In contrast, gamers in the present study exhibited both significantly faster response times and better accuracy than the non-gamers, suggesting broader experience-dependent optimization. Furthermore, prior findings indicate that sex differences in visuomotor response times were significantly reduced as a result of video game-based training (Harwell et al., 2018), emphasizing the role of skill structure in explaining sex differences in their data. Future work using sex-balanced, or targeted samples focusing on female players (Gorbet & Sergio, 2018) will help to refine these findings and further test the generalizability of the observed neural and behavioral adaptations associated with AVGs.

Larger sample sizes would substantially benefit future research. Sensitivity analysis indicated that our study had 80% power to detect effects ≥ *d* ≈ 0.84–0.90 across modalities. Observed effects ranged from *d* = 0.59 to 1.26. While many findings met or exceeded the study’s sensitivity threshold, some effects in the *d* = 0.55–0.75 range fall below the minimum detectable effect size and should be interpreted more carefully with respect to their generalizability beyond this dataset. Furthermore, larger samples would enable more sensitive testing of within-group brain–behavior correlations, making it possible to determine whether observed effects are driven primarily by gamers, non-gamers, or both. These distinctions could clarify group-specific visuomotor strategies and reveal potential subtypes of neural adaptation associated with different gaming subgenres. Additionally, larger cohorts would support a more granular analysis of individual differences and enable more robust comparisons across gaming subgenres (Bediou et al., 2023).

Another limitation is the absence of resting-state fMRI (rs-fMRI) data. Given the success of rcPCA in identifying behaviorally relevant ROIs, it would have been valuable to map these regions onto canonical resting-state networks and compare their connectivity profiles with rs-fMRI data (Zhao et al., 2023). This could offer additional insight into how task-based network dynamics relate to intrinsic functional organization. Unfortunately, resting-state scans were not collected as part of this study, limiting our ability to explore these relationships.

A central assumption of rcPCA’s ROI filtering method used in this study was that the dominant axes of intersubject variance, as identified by PCA, reflect functionally or behaviorally meaningful structure. While this assumption held strongly in the present study, yielding reproducible group differences and behaviorally relevant effects across modalities, it may not generalize to all datasets or neuroimaging metrics. PCA’s linear decomposition may fail to fully capture complex variance structures in metrics characterized by nonlinear relationships.

Our empirical validation supports this view. Spearman rank significance was lost when contributions were limited to the top 20 ROIs per component in dFC receiver and SC connectivity metrics but fully recovered when cumulative contributions across all ROIs were used. This suggests that meaningful contributors may be more diffuse and distributed non-monotonically across components. Such discrepancies may indicate more complex or non-linear variance patterns. Future work could apply nonlinear methods such as kernel PCA, manifold learning, or tensor decompositions to explore whether residual variance reflects interactions or features that escape linear separation. At a minimum, these results emphasize the value of combining linear methods such as PCA with downstream validation to ensure comprehensive coverage of relevant variance space.

ICA may offer further insights in such cases. While ICA is linear, it leverages non-Gaussianity to uncover statistically independent sources of variance, revealing complementary patterns that PCA’s variance-maximizing axes may not capture. Moreover, PCA-derived ROIs could serve as informative priors for joint ICA or other multimodal data fusion frameworks, extending this method’s broader applicability.

Additionally, applying dynamic PCA or tensor decomposition could allow the method to track time-varying or cross-modal shifts in variance structure, offering potential applications in areas such as adaptive brain–computer interfaces (BCIs). Finally, PCA-derived metrics may enhance classification pipelines, supporting both subject-level fingerprinting and group-level identification of neurobiological phenotypes.

Another useful extension would involve calculating the directional skew of each ROI by taking the difference between sender and receiver contributions in dFC (i.e., sender minus receiver). To enable comparisons across subjects and modalities, this difference could be normalized by the total contribution (i.e., sender plus receiver), yielding a normalized score in the range [–1, 1].

Thus, a Directional Skew Index (DI) to capture asymmetries in directed connectivity (dFC) would be defined as

$$DI_{i}=\left(\frac{{\text{Sender}}_{i}-\_{\text{Receiver}}_{\text{i}}}{{\text{Sender}}_{i}+{\text{ Receiver}}_{\text{i}}}\right).$$

A value near +1 would indicate a strong net sender (information source), –1 a strong net receiver (information sink), and 0 a balanced node with symmetrical inflow and outflow. This normalized skew metric could offer deeper insight into causal asymmetries and dynamic role shifts in directed connectivity. By recovering the directional specificity otherwise collapsed in total influence measures, it enhances interpretability and enables a more nuanced characterization of regional dominance in information flow, particularly in the absence of strong priors.

This method offers ample room for future development. Incorporating demographic or clinical covariates (e.g., age, sex, IQ, education, symptom scores) and genetic markers such as single-nucleotide polymorphisms (SNPs) could help contextualize sources of variance and improve generalizability across populations. With further refinement, these tools could contribute to the development of diagnostic or prognostic frameworks in clinical neuroscience. Finally, benchmarking the PCA-derived ROI contributions against alternative feature selection strategies, including ICA, clustering algorithms, or model-derived importance scores from machine learning pipelines, may further clarify when and where this approach provides the most utility. Evaluating robustness across large, multi-site datasets is also critical for assessing generalizability and translational potential. Taken together, the PCA-based ROI selection framework offers a scalable, mathematically principled approach to dimensionality reduction of brain regions to those most informative as measured by contribution to explained variance in neuroimaging analysis. Expanding the framework to include pairwise ROI coupling or grouping ROIs into resting-state or task-defined networks may enable richer interpretations at the level of functional systems rather than individual regions.

### 4.6 Conclusion

This study introduces a novel, data-driven PCA-based method for ROI selection that reveals significant neuroplastic adaptations plausibly driven by action video game (AVG) experience. Our findings provide empirical evidence aligning with Cognitive Resource Reallocation (CRR). Our findings show that AVG experience may promote more efficient visuomotor decision making through top–down cognitive clarity, the unobstructed transformation of learned value into goal-directed action, and bottom–up motor readiness. The convergence of these factors would effectively reduce internal conflict, mitigate visuomotor surprise, and enable rapid yet skillful action selection through greater anticipation of multiple outcomes in the face of uncertainty.

These findings deepen our understanding of the neural mechanisms supporting enhanced visuomotor performance in gamers and have broader implications to inform educational strategy, improve learning programs, and better facilitate skill acquisition. The ability to more effectively reduce uncertainty and resolve internal conflict in high-pressure contexts—as observed in gamers—could potentially inform future design of educational programs, rehabilitation protocols, and high-stakes professional training, including in sports, surgery, and military operations. Moreover, these findings may help shape macro-level rehabilitation goals, especially for patients relearning motor coordination and decision making in dynamic environments.

Our rcPCA method offers ample room for growth and future expansion, such as integrating demographic or clinical variables or applying nonlinear approaches such as kernel PCA to capture more complex variance patterns. This work not only demonstrates the utility of data-driven, principled neuroimaging methods but also provides a foundation for research aimed at enhancing human performance through targeted adaptation.

While future work should assess the generalizability and nonlinear potential of rcPCA, the present study offers both a novel analytical tool and meaningful insight into how experience reshapes cognition. It highlights the brain’s remarkable ability to “level up” by repeatedly confronting and striving to overcome cognitively demanding challenges. Consistent with CRR, these challenges may become less effortful over time, reducing uncertainty and internal conflict, and thereby enabling more efficient, high-performance behavior. This study not only supports that action video game play reflects targeted neuroplasticity but also offers a potentially generalizable blueprint for cognitive optimization through resource reallocation.

## Supplementary Material

Supplementary Material

## Data Availability

All data that support the findings of the study as well as the custom analysis scripts are given in OSF: https://osf.io/9ank7

## References

[IMAG.a.1090-b1] Adhikari, B. M., Jahanshad, N., Shukla, D., Glahn, D. C., Blangero, J., Fox, P. T., Reynolds, R. C., Cox, R. W., Fieremans, E., Veraart, J., Novikov, D. S., Nichols, T. E., Hong, L. E., Thompson, P. M., & Kochunov, P. (2018). Comparison of heritability estimates on resting state fMRI connectivity phenotypes using the ENIGMA analysis pipeline. Hum Brain Mapp, 39(12), 4893–4902. 10.1002/hbm.24331 30052318 PMC6218292

[IMAG.a.1090-b2] Adhikari, B. M., Jahanshad, N., Shukla, D., Glahn, D. C., Blangero, J., Reynolds, R. C., Cox, R. W., Fieremans, E., Veraart, J., Novikov, D. S., Nichols, T. E., Hong, L. E., Thompson, P. M., & Kochunov, P. (2018). Heritability estimates on resting state fMRI data using ENIGMA analysis pipeline. Pac Symp Biocomput, 23, 307–318. https://www.ncbi.nlm.nih.gov/pubmed/29218892 29218892 PMC5728672

[IMAG.a.1090-b3] Adhikari, B. M., Jahanshad, N., Shukla, D., Turner, J., Grotegerd, D., Dannlowski, U., Kugel, H., Engelen, J., Dietsche, B., Krug, A., Kircher, T., Fieremans, E., Veraart, J., Novikov, D. S., Boedhoe, P. S. W., van der Werf, Y. D., van den Heuvel, O. A., Ipser, J., Uhlmann, A., … Kochunov, P. (2019). A resting state fMRI analysis pipeline for pooling inference across diverse cohorts: An ENIGMA rs-fMRI protocol. Brain Imaging Behav, 13(5), 1453–1467. 10.1007/s11682-018-9941-x 30191514 PMC6401353

[IMAG.a.1090-b4] Aurelian, L., Warnock, K. T., Balan, I., Puche, A., & June, H. (2016). TLR4 signaling in VTA dopaminergic neurons regulates impulsivity through tyrosine hydroxylase modulation. Transl Psychiatry, 6(5), e815–e815. 10.1038/tp.2016.72 27187237 PMC5727490

[IMAG.a.1090-b5] Barbot, A., Park, W. J., Ng, C. J., Zhang, R. Y., Huxlin, K. R., Tadin, D., & Yoon, G. (2021). Functional reallocation of sensory processing resources caused by long-term neural adaptation to altered optics. Elife, 10, e58734. 10.7554/eLife.58734 33616034 PMC7963487

[IMAG.a.1090-b6] Basile, G. A., Quartu, M., Bertino, S., Serra, M. P., Boi, M., Bramanti, A., Anastasi, G. P., Milardi, D., & Cacciola, A. (2021). Red nucleus structure and function: From anatomy to clinical neurosciences. Brain Struct Funct, 226(1), 69–91. 10.1007/s00429-020-02171-x 33180142 PMC7817566

[IMAG.a.1090-b89] Bavelier, D., Achtman, R. L., Mani, M., & Föcker, J. (2012). Neural bases of selective attention in action video game players. Vision Research, 61, 132–143. 10.1016/j.visres.2011.08.007 PMC3260403 21864560

[IMAG.a.1090-b7] Bavelier, D., Bediou, B., & Green, C. S. (2018). Expertise and generalization: Lessons from action video games. Curr Opin Behav Sci, 20, 169–173. 10.1016/j.cobeha.2018.01.012

[IMAG.a.1090-b8] Bavelier, D., & Green, C. S. (2019). Enhancing attentional control: Lessons from action video games. Neuron, 104(1), 147–163. 10.1016/j.neuron.2019.09.031 31600511

[IMAG.a.1090-b9] Becker, J. T., Mintun, M. A., Aleva, K., Wiseman, M. B., Nichols, T., & DeKosky, S. T. (1996). Compensatory reallocation of brain resources supporting verbal episodic memory in Alzheimer’s disease. Neurology, 46(3), 692–700. 10.1212/wnl.46.3.692 8618669

[IMAG.a.1090-b10] Bediou, B., Rodgers, M. A., Tipton, E., Mayer, R. E., Green, C. S., & Bavelier, D. (2023). Effects of action video game play on cognitive skills: A meta-analysis. Technol Mind Behav, 4(1). 10.1037/tmb0000102

[IMAG.a.1090-b11] Benjamini, Y., & Hochberg, Y. (1995). Controlling the false discovery rate: A practical and powerful approach to multiple testing. J R Stat Soc Series B Method, 57(1), 289–300. 10.1111/j.2517-6161.1995.tb02031.x

[IMAG.a.1090-b90] Bertoni, S., Franceschini, S., Mancarella, M., Puccio, G., Ronconi, L., Marsicano, G., Gori, S., Campana, G., & Facoetti, A. (2024). Action video games and posterior parietal cortex neuromodulation enhance both attention and reading in adults with developmental dyslexia. Cerebral Cortex, 34(4), bhae152. 10.1093/cercor/bhae152 38610090

[IMAG.a.1090-b12] Bianco, V., Berchicci, M., Quinzi, F., Perri, R. L., Spinelli, D., & Di Russo, F. (2020). Females are more proactive, males are more reactive: Neural basis of the gender-related speed/accuracy trade-off in visuo-motor tasks. Brain Struct Funct, 225(1), 187–201. 10.1007/s00429-019-01998-3 31797033

[IMAG.a.1090-b13] Blihar, D., Delgado, E., Buryak, M., Gonzalez, M., & Waechter, R. (2020). A systematic review of the neuroanatomy of dissociative identity disorder. Eur J Trauma Dissoc, 4(3), 100148. 10.1016/j.ejtd.2020.100148 33433297

[IMAG.a.1090-b14] Borodovitsyna, O., Duffy, B. C., Pickering, A. E., & Chandler, D. J. (2020). Anatomically and functionally distinct locus coeruleus efferents mediate opposing effects on anxiety-like behavior. Neurobiol Stress, 13, 100284. 10.1016/j.ynstr.2020.100284 33344735 PMC7739179

[IMAG.a.1090-b15] Brilliant, T. D., Nouchi, R., & Kawashima, R. (2019). Does video gaming have impacts on the brain: Evidence from a systematic review. Brain Sci, 9(10), 251. 10.3390/brainsci9100251 31557907 PMC6826942

[IMAG.a.1090-b16] Brockett, A. T., Hricz, N. W., Tennyson, S. S., Bryden, D. W., & Roesch, M. R. (2020). Neural signals in red nucleus during reactive and proactive adjustments in behavior. J Neurosci, 40(24), 4715–4726. 10.1523/jneurosci.2775-19.2020 32376779 PMC7294803

[IMAG.a.1090-b17] Brockett, A. T., & Roesch, M. R. (2021). Chapter Ten - Anterior cingulate cortex and adaptive control of brain and behavior. In A. T. Brockett, L. M. Amarante, M. Laubach, & M. R. Roesch (Eds.), International review of neurobiology (Vol. 158, pp. 283–309). Academic Press. 10.1016/bs.irn.2020.11.013 33785148

[IMAG.a.1090-b18] Buhusi, C. V., & Meck, W. H. (2009). Relative time sharing: New findings and an extension of the resource allocation model of temporal processing. Philos Trans R Soc Lond B Biol Sci, 364(1525), 1875–1885. 10.1098/rstb.2009.0022 19487190 PMC2685821

[IMAG.a.1090-b19] Burgess, N., & O’Keefe, J. (2003). Neural representations in human spatial memory. Trends Cogn Sci, 7(12), 517–519. 10.1016/j.tics.2003.10.014 14643363

[IMAG.a.1090-b20] Cahill, K., & Dhamala, M. (2025). Structurally constrained functional connectivity reveals efficient visuomotor decision-making mechanisms in action video gamers. bioRxiv, 2025.2006.2013.659455. 10.1101/2025.06.13.659455 PMC12686510 41360855

[IMAG.a.1090-b21] Cahill, K., Jordan, T., & Dhamala, M. (2024). Connectivity in the dorsal visual stream is enhanced in action video game players. Brain Sci, 14(12), 1206. 10.3390/brainsci14121206 39766405 PMC11674965

[IMAG.a.1090-b22] Calhoun, V. D., Adali, T., Pearlson, G. D., & Pekar, J. J. (2001). A method for making group inferences from functional MRI data using independent component analysis. Hum Brain Mapp, 14(3), 140–151. 10.1002/hbm.1048 11559959 PMC6871952

[IMAG.a.1090-b23] Calhoun, V. D., Liu, J., & Adali, T. (2009). A review of group ICA for fMRI data and ICA for joint inference of imaging, genetic, and ERP data. Neuroimage, 45(1 Suppl), S163–S172. 10.1016/j.neuroimage.2008.10.057 19059344 PMC2651152

[IMAG.a.1090-b24] Caligiore, D., Pezzulo, G., Baldassarre, G., Bostan, A. C., Strick, P. L., Doya, K., Helmich, R. C., Dirkx, M., Houk, J., Jörntell, H., Lago-Rodriguez, A., Galea, J. M., Miall, R. C., Popa, T., Kishore, A., Verschure, P. F. M. J., Zucca, R., & Herreros, I. (2017). Consensus paper: Towards a systems-level view of cerebellar function: The interplay between cerebellum, basal ganglia, and cortex. Cerebellum, 16(1), 203–229. 10.1007/s12311-016-0763-3 26873754 PMC5243918

[IMAG.a.1090-b25] Cavanna, A. E., & Trimble, M. R. (2006). The precuneus: A review of its functional anatomy and behavioural correlates. Brain, 129(Pt 3), 564–583. 10.1093/brain/awl004 16399806

[IMAG.a.1090-b26] Chen, W., Liang, J., Wu, Q., & Han, Y. (2024). Anterior cingulate cortex provides the neural substrates for feedback-driven iteration of decision and value representation. Nat Commun, 15(1), 6020. 10.1038/s41467-024-50388-9 39019943 PMC11255269

[IMAG.a.1090-b27] Cox, R. W. (1996). AFNI: Software for analysis and visualization of functional magnetic resonance neuroimages. Comput Biomed Res, 29(3), 162–173. 10.1006/cbmr.1996.0014 8812068

[IMAG.a.1090-b28] Cox, R. W., & Hyde, J. S. (1997). Software tools for analysis and visualization of fMRI data. NMR Biomed, 10(4–5), 171–178. 10.1002/(sici)1099-1492(199706/08)10:4/5<171::aid-nbm453>3.0.co;2-l 9430344

[IMAG.a.1090-b29] Derosiere, G., Thura, D., Cisek, P., & Duque, J. (2021). Trading accuracy for speed over the course of a decision. J Neurophysiol, 126(2), 361–372. 10.1152/jn.00038.2021 34191623

[IMAG.a.1090-b30] Dhamala, M., Rangarajan, G., & Ding, M. (2008). Analyzing information flow in brain networks with nonparametric Granger causality. Neuroimage, 41(2), 354–362. 10.1016/j.neuroimage.2008.02.020 18394927 PMC2685256

[IMAG.a.1090-b31] Drugowitsch, J., DeAngelis, G. C., Angelaki, D. E., & Pouget, A. (2015). Tuning the speed-accuracy trade-off to maximize reward rate in multisensory decision-making. Elife, 4, e06678. 10.7554/eLife.06678 26090907 PMC4487075

[IMAG.a.1090-b96] Föcker, J., Cole, D., Beer, A. L., & Bavelier, D. (2018). Neural bases of enhanced attentional control: Lessons from action video game players. Brain and Behavior, 8(7), e01019. 10.1002/brb3.1019 PMC6043695 29920981

[IMAG.a.1090-b33] Fotiadis, P., Parkes, L., Davis, K. A., Satterthwaite, T. D., Shinohara, R. T., & Bassett, D. S. (2024). Structure–function coupling in macroscale human brain networks. Nat Rev Neurosci, 25(10), 688–704. 10.1038/s41583-024-00846-6 39103609

[IMAG.a.1090-b97] Franceschini, S., Puccio, G., Bertoni, S., Gori, S., Mascheretti, S., Fusina, F., Angrilli, A., & Facoetti, A. (2025). The benefits of playing action-like video games on salience processing. International Journal of Human-Computer Interaction, 41(7), 4101–4114. 10.1080/10447318.2024.2346693

[IMAG.a.1090-b34] Friston, K. (2010). The free-energy principle: A unified brain theory? Nat Rev Neurosci, 11(2), 127–138. 10.1038/nrn2787 20068583

[IMAG.a.1090-b35] Friston, K., & Kiebel, S. (2009). Predictive coding under the free-energy principle. Philos Trans R Soc Lond B Biol Sci, 364(1521), 1211–1221. 10.1098/rstb.2008.0300 19528002 PMC2666703

[IMAG.a.1090-b36] Glass, B. D., Maddox, W. T., & Love, B. C. (2013). Real-time strategy game training: Emergence of a cognitive flexibility trait. PLoS One, 8(8), e70350. 10.1371/journal.pone.0070350 23950921 PMC3737212

[IMAG.a.1090-b37] Goodale, M. A., & Milner, A. D. (1992). Separate visual pathways for perception and action. Trends Neurosci, 15(1), 20–25. 10.1016/0166-2236(92)90344-8 1374953

[IMAG.a.1090-b38] Gorbet, D. J., & Sergio, L. E. (2018). Move faster, think later: Women who play action video games have quicker visually-guided responses with later onset visuomotor-related brain activity. PLoS One, 13(1), e0189110. 10.1371/journal.pone.0189110 29364891 PMC5783344

[IMAG.a.1090-b39] Granek, J. A., Gorbet, D. J., & Sergio, L. E. (2010). Extensive video-game experience alters cortical networks for complex visuomotor transformations. Cortex, 46(9), 1165–1177. 10.1016/j.cortex.2009.10.009 20060111

[IMAG.a.1090-b40] Green, C. S., & Bavelier, D. (2006). Effect of action video games on the spatial distribution of visuospatial attention. J Exp Psychol Hum Percept Perform, 32(6), 1465–1478. 10.1037/0096-1523.32.6.1465 17154785 PMC2896828

[IMAG.a.1090-b41] Green, C. S., & Bavelier, D. (2007). Action-video-game experience alters the spatial resolution of vision. Psychol Sci, 18(1), 88–94. 10.1111/j.1467-9280.2007.01853.x 17362383 PMC2896830

[IMAG.a.1090-b91] Green, C. S., & Bavelier, D. (2012). Learning, attentional control, and action video games. Current Biology, 22(6), R197–R206. 10.1016/j.cub.2012.02.012 22440805 PMC3461277

[IMAG.a.1090-b92] Gozli, D. G., Bavelier, D., & Pratt, J. (2014). The effect of action video game playing on sensorimotor learning: Evidence from a movement tracking task. Human Movement Science, 38, 152–162. 10.1016/j.humov.2014.09.004 25318081

[IMAG.a.1090-b42] Hahn, B., Ross, T. J., & Stein, E. A. (2006). Neuroanatomical dissociation between bottom–up and top–down processes of visuospatial selective attention. Neuroimage, 32(2), 842–853. 10.1016/j.neuroimage.2006.04.177 16757180 PMC2652125

[IMAG.a.1090-b43] Harwell, K. W., Boot, W. R., & Ericsson, K. A. (2018). Looking behind the score: Skill structure explains sex differences in skilled video game performance. PLoS One, 13(5), e0197311. 10.1371/journal.pone.0197311 29847565 PMC5976164

[IMAG.a.1090-b44] Herlin, B., Navarro, V., & Dupont, S. (2021). The temporal pole: From anatomy to function—A literature appraisal. J Chem Neuroanat, 113, 101925. 10.1016/j.jchemneu.2021.101925 33582250

[IMAG.a.1090-b93] Howard, J., Bowden, V. K., & Visser, T. (2023). Do action video games make safer drivers? The effects of video game experience on simulated driving performance. Transportation Research Part F: Traffic Psychology and Behaviour, 97, 170–180. 10.1016/j.trf.2023.07.006

[IMAG.a.1090-b94] Huang, H., & Cheng, C. (2022). The benefits of video games on brain cognitive function: A systematic review of functional magnetic resonance imaging studies. Applied Sciences, 12(11), 5561. https://www.mdpi.com/2076-3417/12/11/5561

[IMAG.a.1090-b45] Huff, T., Mahabadi, N., & Tadi, P. (2025). Neuroanatomy, visual cortex. In StatPearls. https://www.ncbi.nlm.nih.gov/pubmed/29494110 29494110

[IMAG.a.1090-b46] Ide, J. S., & Li, C. S. (2011). A cerebellar thalamic cortical circuit for error-related cognitive control. Neuroimage, 54(1), 455–464. 10.1016/j.neuroimage.2010.07.042 20656038 PMC2962720

[IMAG.a.1090-b47] Jenkinson, M., Beckmann, C. F., Behrens, T. E., Woolrich, M. W., & Smith, S. M. (2012). Fsl. Neuroimage, 62(2), 782–790. 10.1016/j.neuroimage.2011.09.015 21979382

[IMAG.a.1090-b48] Jolliffe, I. T., & Cadima, J. (2016). Principal component analysis: A review and recent developments. Philos Trans A Math Phys Eng Sci, 374(2065), 20150202. 10.1098/rsta.2015.0202 26953178 PMC4792409

[IMAG.a.1090-b49] Jordan, T., & Dhamala, M. (2022a). Enhanced dorsal attention network to salience network interaction in video gamers during sensorimotor decision-making tasks. Brain Connect, 13(2), 97–106. 10.1089/brain.2021.0193 36053714

[IMAG.a.1090-b50] Jordan, T., & Dhamala, M. (2022b). Video game players have improved decision-making abilities and enhanced brain activities. Neuroimage Rep, 2(3), 100112. 10.1016/j.ynirp.2022.100112 40567307 PMC12172764

[IMAG.a.1090-b51] Kravitz, D. J., Saleem, K. S., Baker, C. I., & Mishkin, M. (2011). A new neural framework for visuospatial processing. Nat Rev Neurosci, 12(4), 217–230. 10.1038/nrn3008 21415848 PMC3388718

[IMAG.a.1090-b52] Krimmel, S. R., Laumann, T. O., Chauvin, R. J., Hershey, T., Roland, J. L., Shimony, J. S., Willie, J. T., Norris, S. A., Marek, S., A, N. V., Wang, A., Monk, J., Scheidter, K. M., Whiting, F. I., Ramirez-Perez, N., Metoki, A., Baden, N. J., Kay, B. P., Siegel, J. S., … Dosenbach, N. U. F. (2025). The human brainstem’s red nucleus was upgraded to support goal-directed action. Nat Commun, 16(1), 3398. 10.1038/s41467-025-58172-z 40210909 PMC11986128

[IMAG.a.1090-b53] Kucukboyaci, N. E., Kemmotsu, N., Leyden, K. M., Girard, H. M., Tecoma, E. S., Iragui, V. J., & McDonald, C. R. (2014). Integration of multimodal MRI data via PCA to explain language performance. NeuroImage Clin, 5, 197–207. 10.1016/j.nicl.2014.05.006 25068109 PMC4110349

[IMAG.a.1090-b98] Kühn, S., & Gallinat, J. (2014). Amount of lifetime video gaming is positively associated with entorhinal, hippocampal and occipital volume. Molecular Psychiatry, 19(7), 842–847. 10.1038/mp.2013.100 23958958

[IMAG.a.1090-b99] Kühn, S., Gallinat, J., & Mascherek, A. (2019). Effects of computer gaming on cognition, brain structure, and function: A critical reflection on existing literature. Dialogues in Clinical Neuroscience, 21(3), 319–330. 10.31887/DCNS.2019.21.3/skuehn 31749656 PMC6829166

[IMAG.a.1090-b100] Kühn, S., Gleich, T., Lorenz, R. C., Lindenberger, U., & Gallinat, J. (2014). Playing Super Mario induces structural brain plasticity: Gray matter changes resulting from training with a commercial video game. Molecular Psychiatry, 19(2), 265–271. 10.1038/mp.2013.120 24166407

[IMAG.a.1090-b101] Kühn, S., Lorenz, R., Banaschewski, T., Barker, G. J., Büchel, C., Conrod, P. J., Flor, H., Garavan, H., Ittermann, B., Loth, E., Mann, K., Nees, F., Artiges, E., Paus, T., Rietschel, M., Smolka, M. N., Ströhle, A., Walaszek, B., Schumann, G., … Gallinat, J. (2014). Positive association of video game playing with left frontal cortical thickness in adolescents. PLoS One, 9(3), e91506. 10.1371/journal.pone.0091506 PMC3954649 24633348

[IMAG.a.1090-b54] Lee, M. H., Kim, N., Yoo, J., Kim, H.-K., Son, Y.-D., Kim, Y.-B., Oh, S. M., Kim, S., Lee, H., Jeon, J. E., & Lee, Y. J. (2021). Multitask fMRI and machine learning approach improve prediction of differential brain activity pattern in patients with insomnia disorder. Sci Rep, 11(1), 9402. 10.1038/s41598-021-88845-w 33931676 PMC8087661

[IMAG.a.1090-b102] Lewandowska, P., Jakubowska, N., Hryniewicz, N., Prusinowski, R., Kossowski, B., Brzezicka, A., & Kowalczyk-Grębska, N. (2022). Association between real-time strategy video game learning outcomes and pre-training brain white matter structure: Preliminary study. Scientific Reports, 12(1), 20741. 10.1038/s41598-022-25099-0 PMC9715544 36456870

[IMAG.a.1090-b55] Li, Z., Tong, L., Zeng, Y., Pei, C., & Yan, B. (2025). Dynamic resource allocation strategies in the human brain under cognitive overload: Evidence from time-varying brain network analysis. Cereb Cortex, 35(3), bhaf048. 10.1093/cercor/bhaf048 40152001

[IMAG.a.1090-b103] Lynch, J., Aughwane, P., & Hammond, T. M. (2010). Video games and surgical ability: A literature review. Journal of Surgical Education, 67(3), 184–189. 10.1016/j.jsurg.2010.02.010 20630431

[IMAG.a.1090-b104] Mancarella, M., Antzaka, A., Bertoni, S., Facoetti, A., & Lallier, M. (2022). Enhanced disengagement of auditory attention and phonological skills in action video gamers. Computers in Human Behavior, 135, 107344. 10.1016/j.chb.2022.107344

[IMAG.a.1090-b56] Mathew, J., Masson, G. S., & Danion, F. R. (2020). Sex differences in visuomotor tracking. Sci Rep, 10(1), 11863. 10.1038/s41598-020-68069-0 32681071 PMC7368072

[IMAG.a.1090-b57] McKnight, P. E., & Najab, J. (2010). Mann-Whitney U test. In The Corsini encyclopedia of psychology (pp. 1-1). 10.1002/9780470479216.corpsy0524

[IMAG.a.1090-b58] Media & Entertainment: Video Games Sector. (2024). https://www.trade.gov/media-entertainment-video-games-sector

[IMAG.a.1090-b59] Mishkin, M., Ungerleider, L. G., & Macko, K. A. (1983). Object vision and spatial vision: Two cortical pathways. Trends Neurosci, 6, 414–417. 10.1016/0166-2236(83)90190-X

[IMAG.a.1090-b60] Monosov, I. E. (2017). Anterior cingulate is a source of valence-specific information about value and uncertainty. Nat Commun, 8(1), 134. 10.1038/s41467-017-00072-y 28747623 PMC5529456

[IMAG.a.1090-b61] Monosov, I. E., Haber, S. N., Leuthardt, E. C., & Jezzini, A. (2020). Anterior cingulate cortex and the control of dynamic behavior in primates. Curr Biol, 30(23), R1442–R1454. 10.1016/j.cub.2020.10.009 33290716 PMC8197026

[IMAG.a.1090-b62] Mushtaq, F., Bland, A. R., & Schaefer, A. (2011). Uncertainty and cognitive control. Front Psychol, 2, 249. 10.3389/fpsyg.2011.00249 22007181 PMC3184613

[IMAG.a.1090-b63] Mwangi, B., Tian, T. S., & Soares, J. C. (2014). A review of feature reduction techniques in neuroimaging. Neuroinformatics, 12(2), 229–244. 10.1007/s12021-013-9204-3 24013948 PMC4040248

[IMAG.a.1090-b107] Orvis, K., Moore, J., Belanich, J., Murphy, J., & Horn, D. (2010). Are soldiers gamers? Videogame usage among soldiers and implications for the effective use of serious videogames for military training. Military Psychology, 22(2), 143–157. 10.1080/08995600903417225

[IMAG.a.1090-b64] Palaus, M., Marron, E. M., Viejo-Sobera, R., & Redolar-Ripoll, D. (2017). Neural basis of video gaming: A systematic review. Front Human Neurosci, 11, 248. 10.3389/fnhum.2017.00248 PMC5438999 28588464

[IMAG.a.1090-b65] Popa, L. S., & Ebner, T. J. (2019). Cerebellum, predictions and errors. Front Cell Neurosci, 12, 524. 10.3389/fncel.2018.00524 30697149 PMC6340992

[IMAG.a.1090-b108] Puderbaugh, M., & Emmady, P. D. (2024). Neuroplasticity. In StatPearls [Internet]. StatPearls Publishing. https://www.ncbi.nlm.nih.gov/books/NBK557811/32491743

[IMAG.a.1090-b66] Renier, L. A., Anurova, I., De Volder, A. G., Carlson, S., VanMeter, J., & Rauschecker, J. P. (2010). Preserved functional specialization for spatial processing in the middle occipital gyrus of the early blind. Neuron, 68(1), 138–148. 10.1016/j.neuron.2010.09.021 20920797 PMC2951740

[IMAG.a.1090-b67] Revell, A. Y., Silva, A. B., Arnold, T. C., Stein, J. M., Das, S. R., Shinohara, R. T., Bassett, D. S., Litt, B., & Davis, K. A. (2022). A framework For brain atlases: Lessons from seizure dynamics. Neuroimage, 254, 118986. 10.1016/j.neuroimage.2022.118986 35339683 PMC9342687

[IMAG.a.1090-b68] Rolls, E. T. (2023). Emotion, motivation, decision-making, the orbitofrontal cortex, anterior cingulate cortex, and the amygdala. Brain Struct Funct, 228(5), 1201–1257. 10.1007/s00429-023-02644-9 37178232 PMC10250292

[IMAG.a.1090-b69] Rolls, E. T., Huang, C.-C., Lin, C.-P., Feng, J., & Joliot, M. (2020). Automated anatomical labelling atlas 3. Neuroimage, 206, 116189. 10.1016/j.neuroimage.2019.116189 31521825

[IMAG.a.1090-b70] Rudebeck, P. H., & Rich, E. L. (2018). Orbitofrontal cortex. Curr Biol, 28(18), R1083–R1088. 10.1016/j.cub.2018.07.018 30253144 PMC9253859

[IMAG.a.1090-b71] Schrauwen, I., Corneveaux, A. S., Peden, J., Turk, M., De Both, M., Richholt, R., Mueller, M., Langbaum, J., Reiman, E., Caselli, R., Coleman, P., Barnes, C., Glisky, E., Ryan, L., Huentelman, M., & Jason. (2014). Between ages 18–85, men exhibit faster reaction times to a visual stimulus. Be a part of our research study into brain function at mindcrowd.org. In: figshare. 10.1038/s41514-021-00067-6

[IMAG.a.1090-b72] Seidler, R. D., Kwak, Y., Fling, B. W., & Bernard, J. A. (2013). Neurocognitive mechanisms of error-based motor learning. Adv Exp Med Biol, 782, 39–60. 10.1007/978-1-4614-5465-6_3 23296480 PMC3817858

[IMAG.a.1090-b73] Shi, W., Meisner, O. C., Blackmore, S., Jadi, M. P., Nandy, A. S., & Chang, S. W. C. (2023). The orbitofrontal cortex: A goal-directed cognitive map framework for social and non-social behaviors. Neurobiol Learn Mem, 203, 107793. 10.1016/j.nlm.2023.107793 37353191 PMC10527225

[IMAG.a.1090-b74] Smith, S. M., Jenkinson, M., Woolrich, M. W., Beckmann, C. F., Behrens, T. E., Johansen-Berg, H., Bannister, P. R., De Luca, M., Drobnjak, I., Flitney, D. E., Niazy, R. K., Saunders, J., Vickers, J., Zhang, Y., De Stefano, N., Brady, J. M., & Matthews, P. M. (2004). Advances in functional and structural MR image analysis and implementation as FSL. Neuroimage, 23 Suppl 1, S208–S219. 10.1016/j.neuroimage.2004.07.051 15501092

[IMAG.a.1090-b75] Sonne, J., Reddy, V., & Beato, M. R. (2025). Neuroanatomy, substantia nigra. In StatPearls [Internet] (Updated 2024 Sep 10 ed.). StatPearls Publishing. https://www.ncbi.nlm.nih.gov/books/NBK536995/30725680

[IMAG.a.1090-b76] Storey, J. D., & Tibshirani, R. (2003). Statistical significance for genomewide studies. Proc Natl Acad Sci U S A, 100(16), 9440–9445. 10.1073/pnas.1530509100 12883005 PMC170937

[IMAG.a.1090-b105] Tulloch, K., & Pammer, K. (2019). Tablet computer games to measure dorsal stream performance in good and poor readers. Neuropsychologia, 130, 92–99. 10.1016/j.neuropsychologia.2018.07.019 30030193

[IMAG.a.1090-b106] Vedamurthy, I., Nahum, M., Bavelier, D., & Levi, D. M. (2015). Mechanisms of recovery of visual function in adult amblyopia through a tailored action video game. Scientific Reports, 5(1), 8482. 10.1038/srep08482 PMC4894407 25719537

[IMAG.a.1090-b77] Veraart, J., Novikov, D. S., Christiaens, D., Ades-Aron, B., Sijbers, J., & Fieremans, E. (2016). Denoising of diffusion MRI using random matrix theory. Neuroimage, 142, 394–406. 10.1016/j.neuroimage.2016.08.016 27523449 PMC5159209

[IMAG.a.1090-b78] Voyer, D., Voyer, S. D., & Saint-Aubin, J. (2017). Sex differences in visual-spatial working memory: A meta-analysis. Psychon Bull Rev, 24(2), 307–334. 10.3758/s13423-016-1085-7 27357955

[IMAG.a.1090-b79] Wall, M. E., Andreas, R., & Rocha, L. M. (2002). Singular value decomposition and principal component analysis. arXiv. 10.1007/0-306-47815-3_5

[IMAG.a.1090-b80] Walter, B. L., & Shaikh, A. G. (2014). Midbrain. In M. J. Aminoff & R. B. Daroff (Eds.), Encyclopedia of the neurological sciences (Second Edition) (pp. 28–33). Academic Press. 10.1016/B978-0-12-385157-4.01161-1

[IMAG.a.1090-b81] Woolrich, M. W., Jbabdi, S., Patenaude, B., Chappell, M., Makni, S., Behrens, T., Beckmann, C., Jenkinson, M., & Smith, S. M. (2009). Bayesian analysis of neuroimaging data in FSL. Neuroimage, 45(1 Suppl), S173–186. 10.1016/j.neuroimage.2008.10.055 19059349

[IMAG.a.1090-b82] Wu, S., & Spence, I. (2013). Playing shooter and driving videogames improves top-down guidance in visual search. Atten Percept Psychophys, 75(4), 673–686. 10.3758/s13414-013-0440-2 23460295

[IMAG.a.1090-b83] Yeh, F. C. (2020). Shape analysis of the human association pathways. Neuroimage, 223, 117329. 10.1016/j.neuroimage.2020.117329 32882375 PMC7775618

[IMAG.a.1090-b95] Yeh, F.-C. (2025). DSI Studio: An integrated tractography platform and fiber data hub for accelerating brain research. Nature Methods, 22, 1617–1619. 10.1038/s41592-025-02762-8 PMC12394933 40707713

[IMAG.a.1090-b84] Yeh, F. C., & Tseng, W. Y. (2011). NTU-90: A high angular resolution brain atlas constructed by q-space diffeomorphic reconstruction. Neuroimage, 58(1), 91–99. 10.1016/j.neuroimage.2011.06.021 21704171

[IMAG.a.1090-b85] Yeh, F. C., Verstynen, T. D., Wang, Y., Fernandez-Miranda, J. C., & Tseng, W. Y. (2013). Deterministic diffusion fiber tracking improved by quantitative anisotropy. PLoS One, 8(11), e80713. 10.1371/journal.pone.0080713 24348913 PMC3858183

[IMAG.a.1090-b86] Yeh, F. C., Wedeen, V. J., & Tseng, W. Y. (2010). Generalized q-sampling imaging. IEEE Trans Med Imaging, 29(9), 1626–1635. 10.1109/TMI.2010.2045126 20304721

[IMAG.a.1090-b87] Zhao, W., Makowski, C., Hagler, D. J., Garavan, H. P., Thompson, W. K., Greene, D. J., Jernigan, T. L., & Dale, A. M. (2023). Task fMRI paradigms may capture more behaviorally relevant information than resting-state functional connectivity. Neuroimage, 270, 119946. 10.1016/j.neuroimage.2023.119946 36801369 PMC11037888

[IMAG.a.1090-b88] Zinchenko, A., Geyer, T., & Föcker, J. (2022). The acquisition but not adaptation of contextual memories is enhanced in action video-game players. Comput Hum Behav, 137, 107401. 10.1016/j.chb.2022.107401

