# Supplementary material for: Level up the brain! Novel PCA method reveals key neuroplastic refinements in action video gamers
Source: Imaging Neurosci (Camb). 2026 Jan 16;4:IMAG.a.1090. doi: 10.1162/IMAG.a.1090 (PMC12820803; doi:10.1162/IMAG.a.1090)
Supplement: Supplementary Material [file IMAG.a.1090_supp.pdf]

SUPPLEMENTARY FIGURE 1. AAL 3 Region Parcellation Categories by Major Anatomical Groups

Original numbers in AAL2 for the anterior cingulate cortex (ACC) and thalamus are left empty in AAL3, as those voxels were substituted by the new subdivisions for the Thalamic nuclei and ACC(Rolls et al., 2020). These regions are removed here, and the index has been adjusted accordingly to the correct 166 total parcellations categorized by major anatomical groups.

No.	AAL 3 Region	Category	No.	AAL 3 Region	Category
**1**	Precentral_L	Frontal	**84**	Temporal_Pole_Sup_R	Temporal
**2**	Precentral_R	Frontal	**85**	Temporal_Mid_L	Temporal
**3**	Frontal_Sup_2_L	Frontal	**86**	Temporal_Mid_R	Temporal
**4**	Frontal_Sup_2_R	Frontal	**87**	Temporal_Pole_Mid_L	Temporal
**5**	Frontal_Mid_2_L	Frontal	**88**	Temporal_Pole_Mid_R	Temporal
**6**	Frontal_Mid_2_R	Frontal	**89**	Temporal_Inf_L	Temporal
**7**	Frontal_Inf_Oper_L	Frontal	**90**	Temporal_Inf_R	Temporal
**8**	Frontal_Inf_Oper_R	Frontal	**91**	Cerebellum_Crus1_L	Cerebellum
**9**	Frontal_Inf_Tri_L	Frontal	**92**	Cerebellum_Crus1_R	Cerebellum
**10**	Frontal_Inf_Tri_R	Frontal	**93**	Cerebellum_Crus2_L	Cerebellum
**11**	Frontal_Inf_Orb_2_L	Frontal	**94**	Cerebellum_Crus2_R	Cerebellum
**12**	Frontal_Inf_Orb_2_R	Frontal	**95**	Cerebellum_3_L	Cerebellum
**13**	Rolandic_Oper_L	Frontal	**96**	Cerebellum_3_R	Cerebellum
**14**	Rolandic_Oper_R	Frontal	**97**	Cerebellum_4_5_L	Cerebellum
**15**	Supp_Motor_Area_L	Frontal	**98**	Cerebellum_4_5_R	Cerebellum
**16**	Supp_Motor_Area_R	Frontal	**99**	Cerebellum_6_L	Cerebellum
**17**	Olfactory_L	Frontal	**100**	Cerebellum_6_R	Cerebellum
**18**	Olfactory_R	Frontal	**101**	Cerebellum_7b_L	Cerebellum
**19**	Frontal_Sup_Medial_L	Frontal	**102**	Cerebellum_7b_R	Cerebellum
**20**	Frontal_Sup_Medial_R	Frontal	**103**	Cerebellum_8_L	Cerebellum
**21**	Frontal_Med_Orb_L	Frontal	**104**	Cerebellum_8_R	Cerebellum
**22**	Frontal_Med_Orb_R	Frontal	**105**	Cerebellum_9_L	Cerebellum
**23**	Rectus_L	Frontal	**106**	Cerebellum_9_R	Cerebellum
**24**	Rectus_R	Frontal	**107**	Cerebellum_10_L	Cerebellum
**25**	OFCmed_L	Orbitofrontal	**108**	Cerebellum_10_R	Cerebellum
**26**	OFCmed_R	Orbitofrontal	**109**	Vermis_1_2	Cerebellum
**27**	OFCant_L	Orbitofrontal	**110**	Vermis_3	Cerebellum
**28**	OFCant_R	Orbitofrontal	**111**	Vermis_4_5	Cerebellum
**29**	OFCpost_L	Orbitofrontal	**112**	Vermis_6	Cerebellum
**30**	OFCpost_R	Orbitofrontal	**113**	Vermis_7	Cerebellum
**31**	OFClat_L	Orbitofrontal	**114**	Vermis_8	Cerebellum
**32**	OFClat_R	Orbitofrontal	**115**	Vermis_9	Cerebellum
**33**	Insula_L	Limbic	**116**	Vermis_10	Cerebellum
**34**	Insula_R	Limbic	**117**	Thal_AV_L	Thalamus
**35**	Cingulate_Mid_L	Limbic	**118**	Thal_AV_R	Thalamus

**36**	Cingulate_Mid_R	Limbic	**119**	Thal_LP_L	Thalamus
**37**	Cingulate_Post_L	Limbic	**120**	Thal_LP_R	Thalamus
**38**	Cingulate_Post_R	Limbic	**121**	Thal_VA_L	Thalamus
**39**	Hippocampus_L	Limbic	**122**	Thal_VA_R	Thalamus
**40**	Hippocampus_R	Limbic	**123**	Thal_VL_L	Thalamus
**41**	ParaHippocampal_L	Limbic	**124**	Thal_VL_R	Thalamus
**42**	ParaHippocampal_R	Limbic	**125**	Thal_VPL_L	Thalamus
**43**	Amygdala_L	Limbic	**126**	Thal_VPL_R	Thalamus
**44**	Amygdala_R	Limbic	**127**	Thal_IL_L	Thalamus
**45**	Calcarine_L	Occipital	**128**	Thal_IL_R	Thalamus
**46**	Calcarine_R	Occipital	**129**	Thal_Re_L	Thalamus
**47**	Cuneus_L	Occipital	**130**	Thal_Re_R	Thalamus
**48**	Cuneus_R	Occipital	**131**	Thal_MDm_L	Thalamus
**49**	Lingual_L	Occipital	**132**	Thal_MDm_R	Thalamus
**50**	Lingual_R	Occipital	**133**	Thal_MDl_L	Thalamus
**51**	Occipital_Sup_L	Occipital	**134**	Thal_MDl_R	Thalamus
**52**	Occipital_Sup_R	Occipital	**135**	Thal_LGN_L	Thalamus
**53**	Occipital_Mid_L	Occipital	**136**	Thal_LGN_R	Thalamus
**54**	Occipital_Mid_R	Occipital	**137**	Thal_MGN_L	Thalamus
**55**	Occipital_Inf_L	Occipital	**138**	Thal_MGN_R	Thalamus
**56**	Occipital_Inf_R	Occipital	**139**	Thal_PuI_L	Thalamus
**57**	Fusiform_L	Occipital	**140**	Thal_PuI_R	Thalamus
**58**	Fusiform_R	Occipital	**141**	Thal_PuM_L	Thalamus
**59**	Postcentral_L	Parietal	**142**	Thal_PuM_R	Thalamus
**60**	Postcentral_R	Parietal	**143**	Thal_PuA_L	Thalamus
**61**	Parietal_Sup_L	Parietal	**144**	Thal_PuA_R	Thalamus
**62**	Parietal_Sup_R	Parietal	**145**	Thal_PuL_L	Thalamus
**63**	Parietal_Inf_L	Parietal	**146**	Thal_PuL_R	Thalamus
**64**	Parietal_Inf_R	Parietal	**147**	ACC_sub_L	Limbic
**65**	SupraMarginal_L	Parietal	**148**	ACC_sub_R	Limbic
**66**	SupraMarginal_R	Parietal	**149**	ACC_pre_L	Limbic
**67**	Angular_L	Parietal	**150**	ACC_pre_R	Limbic
**68**	Angular_R	Parietal	**151**	ACC_sup_L	Limbic
**69**	Precuneus_L	Parietal	**152**	ACC_sup_R	Limbic
**70**	Precuneus_R	Parietal	**153**	N_Acc_L	Limbic
**71**	Paracentral_Lobule_L	Parietal	**154**	N_Acc_R	Limbic
**72**	Paracentral_Lobule_R	Parietal	**155**	VTA_L	Limbic
**73**	Caudate_L	Basal Ganglia	**156**	VTA_R	Limbic
**74**	Caudate_R	Basal Ganglia	**157**	SN_pc_L	Limbic
**75**	Putamen_L	Basal Ganglia	**158**	SN_pc_R	Limbic
**76**	Putamen_R	Basal Ganglia	**159**	SN_pr_L	Limbic

**77**	Pallidum_L	Basal Ganglia	**160**	SN_pr_R	Limbic
**78**	Pallidum_R	Basal Ganglia	**161**	Red_N_L	Brainstem
**79**	Heschl_L	Temporal	**162**	Red_N_R	Brainstem
**80**	Heschl_R	Temporal	**163**	LC_L	Brainstem
**81**	Temporal_Sup_L	Temporal	**164**	LC_R	Brainstem
**82**	Temporal_Sup_R	Temporal	**165**	Raphe_D	Brainstem
**83**	Temporal_Pole_Sup_L	Temporal	**166**	Raphe_M	Brainstem

SUPPLEMENTARY FIGURE 2. Group-Level Statistics Across Modalities

Comparisons between action video-game players and non-gamers for functional connectivity (FC), directed functional connectivity (dFC: sender/receiver/total), structural connectivity (SC) metrics, and structure–function coupling (SFC, SdFC). Entries report *p* value, FDR-adjusted *q* value, and Cohen's *d* (positive = favors gamers; negative = favors non-gamers). FDR control used Storey–Tibshirani (ST) for FC and Benjamini–Hochberg (BH) elsewhere; blank *q* cells indicate effects not surviving FDR within that family. Abbreviations: ACC sup = superior anterior cingulate; Thal = thalamus (IL = intralaminar, VL = ventrolateral, LP = lateral posterior); SN pr/pc = substantia nigra pars reticulata/compacta; Red N = red nucleus; LC = locus coeruleus.

**Functional Connectivity (FC)**		**p-value**	**q-value (ST)**	**Cohen's d**
Red N R	Thal Pul R	0.0061	0.02	0.85
Cerebellum 3L	Temporal Pole Sup R	0.0014	0.035	1.12
Cerebellum 3L	Caudate L	0.00093	0.03	1.00
Cerebellum 3L	Thal IL L	0.00085	0.042	-1.26
Cerebellum 3L	SN pr L	0.002	0.042	-1.10
Cerebellum 3L	Thal VL R	0.01	_	-0.76
Cerebellum 3L	Thal VL L	0.03	_	-0.87
**Directed Connectivity (Sender)**		**p-value**	**q-value (BH)**	**Cohen's d**
ACC sup L	Thal VL R	0.0003	0.042	-0.91
ACC sup L	Cerebellum 7b L	0.004	_	-0.65
VTA R	Thal VL R	0.006	_	-0.96
VTA R	Cerebellum 3L	0.012	_	-0.75
**Directed Connectivity (Receiver)**		**p-value**	**q-value (BH)**	**Cohen's d**
Cerebellum 3L	ACC sup L	0.004	_	-1.00
LC R	ACC sup L	0.031	_	-0.83
VTA L	Cerebellum 3L	0.0008	_	-0.91
VTA R	Cerebellum 3L	0.008	_	-0.75
Thal VL R	Cerebellum 3L	0.029	_	-0.61
**Directed Connectivity (Total)**		**p-value**	**q-value (BH)**	**Cohen's d**
ACC sup L	Thal VL R	0.002	_	-0.98
Cerebellum 3L	ACC sup L	0.011	_	-0.87
Red N R	SN pc R	0.024	_	-0.79
Thal LP R	Cerebellum 3L	0.038	_	-0.59
**Structural Connectivity**				
**Fractional Anisotropy (FA)**		**p-value**	**q-value (BH)**	**Cohen's d**
Calcarine L	Occipital Sup L	0.036	_	-0.78
**Axial Diffusivity (AD)**		**p-value**	**q-value (BH)**	**Cohen's d**
Frontal Sup Medial L	Cingulate Mid L	0.0003	0.047	1.16
**Isotropy (ISO)**		**p-value**	**q-value (BH)**	**Cohen's d**
Lingual L	Caudate L	0.0002	0.026	1.22

Lingual R	Cerebellum 6 L	0.0007	0.047	0.96
**Non-Restricted Diffusion (NDRI)**		**p-value**	**q-value (BH)**	**Cohen's d**
Lingual L	Caudate L	0.00008	0.037	0.97
Lingual R	Cerebellum 6 L	0.0007	0.047	0.87
**Structure-Function Coupling**				
**Structure - Function Coupling (SFC)**	**SC Measure**	**p-value**	**q-value (BH)**	**Cohen's d**
Vermis 3	Mean Diffusivity (MD)	0.046	_	0.64
Vermis 9	Mean Length	0.024	_	0.64
Vermis 9	Fractional Anisotropy (FA)	0.049	_	0.55
Cerebellum 10L	Fractional Anisotropy (FA)	0.046	_	0.65
Cerebellum 10L	Quantitative Anisotropy (QA)	0.049	_	0.65
**Structure - Directed Coupling (SdFC , Sender)**	**SC Measure**	**p-value**	**q-value (BH)**	**Cohen's d**
Vermis 7	Axial Diffusivity (AD)	0.002	0.048	-1.00
Vermis 7	Isotropy (ISO)	0.004	_	-0.97
Vermis 7	Restricted Diffusion (RDI)	0.004	_	-0.97
Calcarine R	count	0.038	_	-0.74
Calcarine R	ncount	0.015	_	-0.82
Calcarine R	ncount2	0.023	_	-0.82
Calcarine L	count	0.043	_	-0.59
ACC sup L	ncount	0.02	_	0.82
ACC sup L	ncount2	0.021	_	0.83
Frontal Medial Orb L	count	0.032	_	0.74
Paracentral Lobule R	ncount	0.028	_	-0.67
